# A novel tauopathy model mimicking molecular and spatial aspects of human tau pathology

**DOI:** 10.1093/braincomms/fcae326

**Published:** 2024-09-19

**Authors:** Rin Yanai, Tomoki T Mitani, Etsuo A Susaki, Takeharu Minamihisamatsu, Masafumi Shimojo, Yuri Saito, Hiroshi Mizuma, Nobuhiro Nitta, Daita Kaneda, Yoshio Hashizume, Gen Matsumoto, Kentaro Tanemura, Ming-Rong Zhang, Makoto Higuchi, Hiroki R Ueda, Naruhiko Sahara

**Affiliations:** Advanced Neuroimaging Center, Institute for Quantum Medical Sciences, National Institutes for Quantum Science and Technology, Chiba, 263-8555, Japan; Laboratory for Synthetic Biology, RIKEN BDR, Suita, Osaka, 565-0871, Japan; Department of Systems Biology, Graduate School of Medicine, Osaka University, Suita, Osaka, 565-0871, Japan; Department of Neurology, Graduate School of Medicine, Osaka University, Suita, Osaka, 565-0871, Japan; Laboratory for Synthetic Biology, RIKEN BDR, Suita, Osaka, 565-0871, Japan; Department of Biochemistry and Systems Biomedicine, Graduate School of Medicine, Juntendo University, Tokyo, 113-8421, Japan; Nakatani Biomedical Spatialomics Hub, Graduate School of Medicine, Juntendo University, Tokyo, 113-8421, Japan; Advanced Neuroimaging Center, Institute for Quantum Medical Sciences, National Institutes for Quantum Science and Technology, Chiba, 263-8555, Japan; Advanced Neuroimaging Center, Institute for Quantum Medical Sciences, National Institutes for Quantum Science and Technology, Chiba, 263-8555, Japan; Department of Biochemistry and Systems Biomedicine, Graduate School of Medicine, Juntendo University, Tokyo, 113-8421, Japan; Advanced Neuroimaging Center, Institute for Quantum Medical Sciences, National Institutes for Quantum Science and Technology, Chiba, 263-8555, Japan; Advanced Neuroimaging Center, Institute for Quantum Medical Sciences, National Institutes for Quantum Science and Technology, Chiba, 263-8555, Japan; Department of Neuropathology, Choju Medical Institute, Fukushimura Hospital, Aichi, 441-8124, Japan; Department of Neuropathology, Choju Medical Institute, Fukushimura Hospital, Aichi, 441-8124, Japan; Department of Neurological Disease Control, Osaka Metropolitan University, School of Medicine, Osaka, 545-8585, Japan; Laboratory of Animal Reproduction and Development, Graduate School of Agricultural Science, Tohoku University, Miyagi, 980-8572, Japan; Department of Advanced Nuclear Medicine Science, Institute for Quantum Medical Sciences, National Institutes for Quantum Science and Technology, Chiba, 263-8555, Japan; Advanced Neuroimaging Center, Institute for Quantum Medical Sciences, National Institutes for Quantum Science and Technology, Chiba, 263-8555, Japan; Laboratory for Synthetic Biology, RIKEN BDR, Suita, Osaka, 565-0871, Japan; Department of Systems Biology, Graduate School of Medicine, Osaka University, Suita, Osaka, 565-0871, Japan; Department of Systems Pharmacology, Graduate School of Medicine, The University of Tokyo, Tokyo, 113-0033, Japan; Advanced Neuroimaging Center, Institute for Quantum Medical Sciences, National Institutes for Quantum Science and Technology, Chiba, 263-8555, Japan

**Keywords:** tauopathy, model mouse, tissue clearing, whole brain, tau pathology

## Abstract

Creating a mouse model that recapitulates human tau pathology is essential for developing strategies to intervene in tau-induced neurodegeneration. However, mimicking the pathological features seen in human pathology often involves a trade-off with artificial effects such as unexpected gene insertion and neurotoxicity from the expression system. To overcome these issues, we developed the rTKhomo mouse model by combining a transgenic CaMKII-tTA system with a P301L mutated 1N4R human tau knock-in at the *Rosa26* locus with a C57BL/6J background. This model closely mimics human tau pathology, particularly in the hippocampal CA1 region, showing age-dependent tau accumulation, neuronal loss and neuroinflammation. Notably, whole-brain 3D staining and light-sheet microscopy revealed a spatial gradient of tau deposition from the entorhinal cortex to the hippocampus, similar to the spatial distribution of Braak neurofibrillary tangle staging. Furthermore, [^18^F]PM-PBB3 positron emission tomography imaging enabled the quantification and live monitoring of tau deposition. The rTKhomo mouse model shows potential as a promising next-generation preclinical tool for exploring the mechanisms of tauopathy and for developing interventions targeting the spatial progression of tau pathology.

## Teaser

The authors report that the newly developed tauopathy mouse model, termed rTKhomo, exhibits progressive accumulation of pathological tau, neuronal loss and age-related neuroinflammation. Whole-brain 3D imaging unveiled the distribution of tau pathology along the entorhinal–hippocampal connections. This model will serve as a valuable tool for the intervention of tau-induced disorders.

## Graphical Abstract

**Graphical Abstract:**
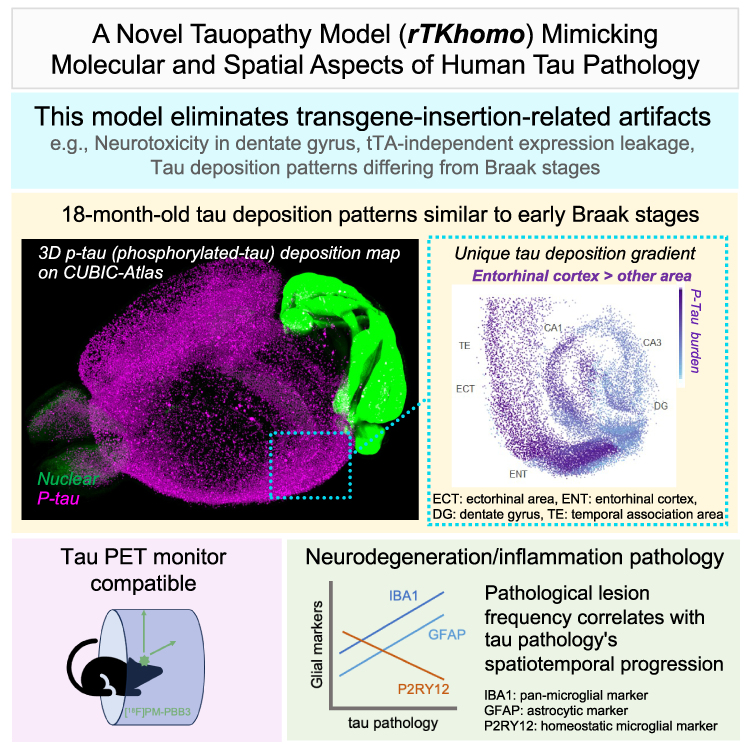

## Introduction

Neurofibrillary tangles (NFTs) composed of hyperphosphorylated tau protein are primarily neuropathological features of a number of neurodegenerative diseases, collectively termed tauopathy.^1^ For the purpose of developing animal models of human tauopathy by showing prominent intracellular deposition of tau protein and associated neuronal loss, several transgenic (tg) mouse lines expressing human tau with or without frontotemporal lobar degeneration (FTLD)-linked mutations have been developed.^2^ Until now, overexpression of non-mutant human tau transgenes has had only limited success in developing animal models with either mature tau pathologies or behaviour abnormality.^3^ It is most likely that FTLD-linked mutations will accelerate NFT formation, other tau-related pathologies and neurodegeneration. In fact, multiple lines of P301L/S mutant tau-expressing tg mice developed neurofibrillary pathology in the central nervous system (CNS) in a promoter-dependent manner.^4-8^

On the other hand, recent studies revealed that transgenes disrupt the coding sequence of endogenous genes, resulting in deletions and/or structural variations at the insertion site.^9^ In the rTg4510 mouse line,^7^ which is derived from cross-breeding with two tg mice, showed deletion mutation of fibroblast growth factor 14 (*Fgf14*) in tau responder tg mice and disruptions of *Vipr2, Wdr60, Esyt2, D430020J02Rik*, *Ncapg2* and *Ptprn2* genes in Tg (CaMKIIa-tTA)1Mmay mice (tTA tg mice).^9^ To avoid unexpected gene disruptions, Gamache *et al*. took a homologous recombination approach with an insertion of human P301L tau into a site downstream of collagen type alpha 1 (*Col1A1*) and observed that the resultant human P301L tau overexpressing mice showed delayed brain atrophy with intact *Fgf14* gene in comparison with the rTg4510 mice.^10^ As for the other model created by homologous recombination that avoids gene disruption, *MAPT* knock-in mice were created by replacing the entire mouse *Mapt* gene with a human ortholog.^11^ In the *MAPT* KI mice, expression of all six tau isoforms was reproducing, but they failed to develop apparent age-dependent tau pathology and neurodegeneration,^12^ perhaps due to the absence of FTLD-linked mutations and relatively lower expression levels of tau protein compared with human tau overexpressing tg mouse lines. Such situations prompted us to develop a novel mouse model, which can induce a major expression of tau without gene deficits and reproduce the pathology of human tauopathy.

Neuroinflammatory responses are another issue that reflects human tauopathy and related neurodegenerative disorders. Activated astrocytes and microglia are well associated with both amyloid and tau pathologies.^13^ During pathogenesis, microglia could become a chronic source of multiple neurotoxic factors, such as tumour necrosis factor-α, nitric oxide, interleukin-1β and reactive oxygen species.^14^ Recent studies suggested that both neuroinflammation and peripheral inflammation could induce vulnerability of the CNS.^15^ Regarding therapeutic interventions, the immunosuppressive drug FK506 has demonstrated the capability to mitigate tauopathy and extend the lifespan of the tauopathy mouse model, PS19, which expresses the P301S mutant tau.^8^ This finding supports therapeutic strategies to prevent disease progression by manipulating neuroinflammatory processes when a potential window of drug efficacy will be resolved. However, attention should be paid to the use of mouse models that may contain genetic engineering defects that could affect tau pathology and neurodegeneration. For example, neuroinflammation and neuronal death in the hippocampal dentate gyrus (DG) due to tTA, which drives the Tet-off system, has been reported in FVB and 129 strains,^16^ strains that are also used in rTg4510 mice. It will be crucial to test anti-inflammatory drugs on precise experimental models, but no model has been reported that avoids all of these problems and develops apparent tau pathology and neurodegeneration.

Since random integration may cause an unstable expression of transgenes and unpredictable phenotypes, it is preferable to insert target genes into a safe locus.^17,18^ The mouse *Rosa26* locus shows ubiquitous transcriptional activity but a loss of this gene is not lethal.^19^ This locus is widely used as a permissive site for targeted placement of transgenes in mice without any effect on animal viability or fertility.^20,21^ Moreover, Rosa26 knock-in reporter mouse models demonstrated that chromatin configurations leading to the transcriptional repression of exogenous transgenes may not be effective at this locus.^22^ Therefore, knock-in of a transgene into the *Rosa26* locus enables locus specificity without any endogenous gene disruptions of transgene expression. In this study, we attempted human tau gene knock-in at the *Rosa26* locus to create a novel tauopathy mouse model that recapitulates promoter-dependent tau expression and tau pathology in the absence of endogenous gene mutations in principle, and that can be deployed for various applications. However, knock-in alone has not been able to reproduce age-dependent NFT-like pathology.^11,12^ Therefore, to induce overexpression, the Rosa26 knock-in mice were cross-bred with CaMKII-tTA tg mice resulting in CaMKII promoter-dependent tau transgene overexpression. Since it was reported that tTA causes mouse strain-dependent neuronal loss in the dentate granule cell layer and behavioural alterations, we chose a congenic C57BL/6J background that was resistant to tTA-induced degeneration for the development of double tg mice.^16^ In this study, we evaluated the utility of this model by analyzing the distribution of tau deposition across the whole brain. Additionally, our data showed age-dependent development of tau pathology and tau-induced neuronal loss in aged mice. In parallel with pathological tau formation, a variety of microglial morphologies (e.g. ramified, rod-shaped, amoeboid) was observed in hippocampal regions at over 15 months of age. Our data indicated that a design that avoids artificial phenotypes as much as possible could develop tauopathy model mice that mimic human pathology more accurately.

## Materials and methods

### Animals

All mice were housed in cages at 25°C in a 12-h light/dark cycle, with ad libitum food and water. All experiments were performed in accordance with the institutional guidelines on the use of laboratory animals and were approved by the National Institutes for Quantum Science and Technology Institutional Animal Care and Use Committees.

**Figure 1:**
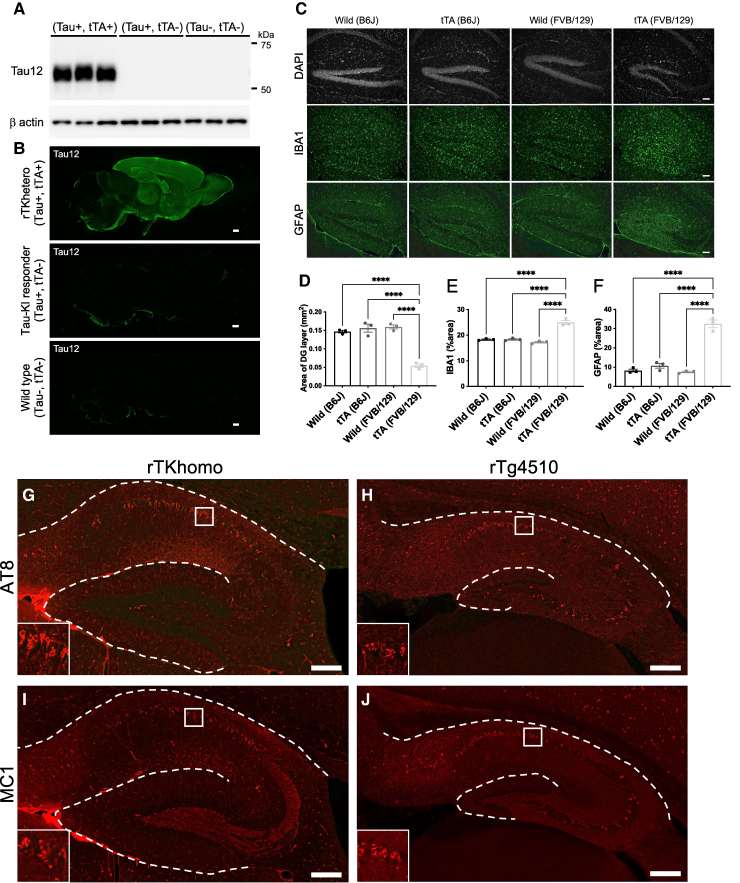
**Elimination of defects reported in rTg4510 and formation of characteristic tau pathology in rTKhetero/homo mice.** (**A**) Western blotting of human tau and β-actin protein in buffer-extractable fractions from 2-month-old rTKhetero mice (Tau+/−, tTA+/−), Tau-KI responder mice (Tau+/−, tTA−/−) and wild type mice (Tau−/−, tTA−/−). Each genotype *n* = 3 female. See Supplementary Fig. 10 for uncropped blots. (**B**) Human tau immunoreactivity labelled by Tau12 antibody in sagittal brain sections from 2-month-old rTKhetero mice, Tau-KI responder mice and wild-type mice. Scale bars = 500 µm. (**C**) DAPI stained, IBA1 immunofluorescence and GFAP immunofluorescence images of dentate gyrus from wild type and tTA tg on the congenic B6 background, and from wild type and tTA tg on 129 × FVB F1 hybrid background. Scale bars = 100 µm. (**D**) Areas of dentate granule cell layers in wild type and tTA tg on the congenic B6 background (*n* = 3 females each), and from wild type and tTA tg on 129 × FVB F1 hybrid background (*n* = 3 females each). Averaged areas were taken from three serial sagittal sections of each mouse. (**E**) Ratios of IBA1-positive signals in the dentate gyri. 3 females per group. (**F**) Ratios of GFAP-positive signals in the dentate gyri. 3 females per group. ****P < 0.0001 (Tukey’s multiple comparison). (**G–J**) Upper panels show AT8 immunofluorescence staining of hippocampus region from 18-month-old rTKhomo (Tau+/+, tTA+/−) mouse (**G**) and 6-month-old rTg4510 mouse (**H**). Lower panels show conformation-specific tau antibody MC1 immunofluorescence staining of hippocampus region from 18-month-old rTKhomo mouse (**I**) and 6-month-old rTg4510 mouse (**J**). Inboxes show tau antibody-positive neurons in pyramidal cell layers of CA1. Scale bars = 200 µm.

To generate human tau-expressing mice, a targeting vector containing P301L mutated human 1N4R tau cDNA with the Tet-responsive *P*_tight_ promoter was designed (Supplementary Fig. 1A). Briefly, the vector was designed for insertion into the intron between Exon1 and Exon2 in the *Rosa26* locus, which was based on the Mouse Genomic Information of the National Center for Biotechnology Information (NCBI, NC_000072). The vector contained the flippase (FLP) recognition target (FRT)-Neomycin resistance gene (NEO)-FRT sequence required for positive selection and diphtheria toxin fragment A (DTA) gene for negative selection (Supplementary Fig. 1A). As for the generation of Tau-KI responders (Supplementary Fig. 1A), establishment of target ES cells, generation of chimera and F1 mice and removal of the neo cassette were conducted by Unitech Co. (Kashiwa City, Japan). Novel human tau overexpressing mice (Rosa26-KI tau (±) expressing mice) were generated by crossing Tau-KI responder mice with CaMKII-tTA (under C57BL/6J stain background) tg mice expressing tetracycline-controlled transactivators from the CaMKII promoter (introduced by Jackson Laboratory, USA) (Fig. 1). Genotyping was performed by PCR with primer sets: (Short Arm forward: 5′-AGGAGTTAAAACTTTCCTTCTGAGC-3′, TRE-tight forward: 5′− ATAAGCAGAGCTCGTTTAGTGAACC-3′, NEO forward: 5′− CTTCCTCGTGCTTTACGGTATC-3′, human tau forward: 5′− GTTTTATTGAGTTCTGAAGGTTGGA-3′, human tau reverse: 5′-ATCAGACTTCTAAGATCAGGAAAGG-3′, CaMKII-tTA forward: 5′-GACCTGGATGCTGACGAAGG-3′, CaMKII-tTA reverse: 5′-GCAGCTCTAATGCGCTGT-3′).

All experimental animals, along with age at dissection, sex, genotype, generation and corresponding figure numbers are listed in Supplementary Table 1.

### Human tissue

Postmortem human brains [two senile dementia of the NFT type (SD-NFT) and three non-demented control subjects, Table 1] were obtained from Fukushimura Hospital. Hippocampus and temporal cortices were examined from each brain specimen. Amyloid histopathology was assessed according to CERAD criteria.^23^ NFT pathology was staged according to Braak staging.^24^ Cases of SD-NFT, a subset of dementia characterized by a number of NFTs in the hippocampal region and the absence of amyloid plaques in the brain, were diagnosed by these criteria.^25,26^ All procedures involving the use of human materials were conducted following performed in accordance with the ethical guidelines of the Institutional Review Boards of Fukushimura Hospital and the National Institutes for Quantum Science and Technology.

**Table 1:** Demographic and pathologic features of human subjects

Case	Pathological diagnosis	Brain regions	Age (yr)	Sex	Braak NFT	CERAD plaque score
SDNFT-1	NFTD	HP, SUB	96	F	III	0
SDNFT-2	NFTD	HP, SUB	96	F	III	A
Control-1	Control	HP, SUB	96	F	II	0
Control-2	Control	HP, SUB	90	F	II	0
Control-3	Control	HP, SUB	77	M	I	0

CERAD, Consortium to Establish a Registry for Alzheimer’s Disease; HP, Hippocampus; F, Female; M, Male; NFT, Neurofibrillary tangle; NFTD, Neurofibrillary tangle-predominant dementia; SDNFT, Senile dementia of the NFT type; SUB, Subiculum.

### Antibodies and compounds

Mouse monoclonal Tau5 and Tau12 antibodies were a kind gift from Dr. Nicholas M. Kanaan (Michigan State University, USA). MC1 and PHF1 antibodies were provided by Dr. Peter Davies (Feinstein Institutes for Medical Research). Rabbit polyclonal mTau antibody specific to rodent tau (aa 118–131) was prepared in our laboratory. Tau46 (4019S, mouse monoclonal, Cell Signaling Technology, USA), HT7 (MN1000, mouse monoclonal, Thermo Fisher Scientific, Waltham, MA), AT8 (MN1020, mouse monoclonal, Thermo Fisher Scientific), β-actin (A1987, mouse monoclonal, Merck Millipore, Burlington, MA), Ms X Neuronal Nuclei (NeuN) (MAB377, mouse monoclonal, Merck Millipore), IBA1 (MABN92, mouse monoclonal, Merck Millipore), IBA1 (019− 19741, rabbit polyclonal, Wako, Japan), P2RY12 (rabbit polyclonal, in house)^27^ and GFAP (2.2B10, rat monoclonal, Zymed, San Francisco, CA) antibodies were used for western blotting and immunohistochemistry.

PM-PBB3, 1-fluoro-3-((2-((1E,3E)-4-(6-(methylamino) pyridine-3-yl) buta-1,3-dien-1-yl) benzo[d]thiazol-6-yl) oxy) propan-2-ol and the tosylate precursor of PM-PBB3 were custom-synthesized (Nard Institute, Japan). The precursor of PM-PBB3 was also provided by APRINOIA Therapeutics Inc (Taiwan). We synthesized and used [^18^F]PM-PBB3 under UV-cut light to avoid photo-isomerization of these compounds as described previously.^28^

### Mouse tissue preparation for western blotting and immunofluorescence staining

Brain extraction was performed according to a previous report.^29^ Briefly, after euthanizing by cervical dislocation, mouse brains were bisected along the midline to produce two hemispheres. The left hemisphere’s cerebral cortex and hippocampus from each animal were rapidly frozen on dry ice and stored at −80°C until use. Tissues were then homogenized in 10 volumes of Tris-buffered saline [TBS: 50 mM Tris/HCl (pH 7.4), 274 mM NaCl, 5 mM KCl, 1% protease inhibitor mixture (Sigma, St. Louis, MO), 1% phosphatase inhibitor cocktail I and II (Sigma) and 1 mM phenylmethylsulfonyl fluoride (PMSF)]. After saving a portion of the homogenates (total homogenate), the remaining homogenates were centrifuged at 27 000 × g for 20 min at 4°C to obtain supernatant (S1) and pellet fractions. Pellets were then homogenized in 5 volumes of high salt/sucrose buffer [0.8 M NaCl, 10% sucrose, 10 mM Tris/HCl, (pH 7.4), 1 mM EGTA, 1 mM PMSF] and centrifuged under the same condition. The supernatants were collected and incubated with sarkosyl (1% final concentration; SERVA Electrophoresis GmbH, Germany) for one hour at 37°C, followed by centrifugation at 150 000 × g for 1 h at 4°C to obtain salt and sarkosyl-extractable (S3) and sarkosyl-insoluble (P3) fractions. The P3 pellet was re-suspended in TE buffer [10 mM Tris/HCl (pH 8.0), 1 mM EDTA] to a volume equivalent to half of the brain specimens used to produce original brain homogenates.

### Western blotting

Fractionated tissue extracts were dissolved in SDS-sample buffer containing β-mercaptoethanol (2.5%). The heat-treated samples (55°C for 15 min) were separated by gel electrophoresis on 10% Tris-glycine SDS-PAGE gels (Nacalai, Japan) and transferred onto nitrocellulose membranes (BioRad Laboratories, Hercules, CA). After blocking with a blocking solution containing 5% nonfat milk and 0.05% Tween-20 in TBS, the membranes were incubated with primary antibodies, such as Tau12 (1:5000), Tau46 (1:2000), AT8 (1:2000) and PHF1 (1:2000) and β-actin (1:2000). After washing with TBS-Tween-20, membranes were incubated with peroxidase-conjugated goat anti-rabbit antibodies (1:5000; Jackson ImmunoResearch, West Grove, PA) or anti-mouse IgG (1:5000; Jackson ImmunoResearch). Bound antibodies were detected using an enhanced chemiluminescence system (ECL PLUS kit; PerkinElmer, Waltham, MA). Western blot immunoreactivity was visualized by Amersham Imager 600 (GE Healthcare). Band intensity quantification was performed with ImageJ (version 1.53, National Institutes of Health, USA) (Supplementary Figs. 2 and 3).

### Immunofluorescence staining

The right hemisphere of each animal was fixed with 4% paraformaldehyde in PBS. These brains were then cryoprotected with 20% sucrose, embedded in OCT compound (Sakura Finetek Japan, Japan) and sliced into 10 or 20-µm sections. For antigen retrieval, cryo-sections were washed three times with PBS or paraffin-sections were deparaffined, incubated in citric acid solution [0.01 M sodium citrate, 0.01 M citric acid (pH 6.0)] at 80°C for 15 min, followed by blocking with blocking solution (4% BSA, 2% horse serum, 0.25% Triton X-100 in PBS). After washing with PBS, sections were incubated with primary antibodies: Tau12 (1:500), HT7 (1:500), AT8 (1:500), MC1 (1:200), NeuN (1:500), IBA1 (Wako, 1:500; Merck, 1:500), P2RY12 (1:5000), GFAP (1:500). After washing with PBS-Tween-20, sections were incubated with fluorescent conjugated secondary antibodies (1:500, Jackson ImmunoResearch). Images were captured by fluorescence microscope (BZ-X710, Keyence, Japan, DM4000 B LED, Leica, Germany) or confocal microscope (LSM880, Carl Zeiss, Germany).

### Whole brain clearing and 3D staining

To clear brain tissue, we adopted CUBIC protocols.^30,31^ CUBIC-L (Tokyo Chemical Industry, Japan) for delipidation and CUBIC-R + (N) (Tokyo Chemical Industry) for RI matching were used. Each brain was stained with primary and secondary antibodies and SYTOX-G (ThermoFisher Scientific) for nuclear staining by modified CUBIC-HistoVIsion protocol.^31^ For detection of tau pathology with 3D immunostaining, we used AT8 (5 µg/brain) as primary antibody and Alexa Fluor 647 AffiniPure Fab Fragment Goat Anti-Mouse IgG1, Fcγ fragment specific (4 µg/brain) (Jackson ImmunoResearch) as secondary antibody. Before imaging, the sample was embedded in a mounting medium with RI matching 1.520.

### Workflow of whole-brain analysis

Whole-brain analysis encompasses four major phases: (i) initial expert visual examination of the raw data and determination of excluded anatomical regions, (ii) data preprocessing, (iii) tau extraction via Gaussian-mean difference, and (iv) whole-brain registration using the CUBIC-Cloud platform (https://cubic-cloud.com), followed by exclusion of specific anatomical regions. The analytical steps were undertaken using a custom code developed with Python 3.6.

#### Expert review of raw data for anatomical region exclusion

The raw data of phospho-Tau (AT8) channel images covering the entire brain were first examined visually, under intense review, by several experts. Due to the identification of non-specific staining consistent with anatomical structures in the ventricular systems and fibre tracts, these areas were predetermined to be excluded from the (iv) analysis.

#### Data preprocessing

Brain data from three 18-month-old rTKhomo mice had a voxel size of 8.3 × 8.3 × 8.3 µm or 6.5 × 6.5 × 8.3 µm. To ensure the consistency of resolution, data originally at 6.5 ×6.5 × 8.3 µm were re-sampled to the standard resolution of 8.3 × 8.3 × 8.3 µm.

#### Tau deposition regions of interest extraction via Gaussian-mean difference

In order to amplify the tau signal relative to the local background intensity, we employed a Gaussian filter with a smaller size (sigma being 0.83, translating to a full-width at half-maximum of approximately 16 × 16 × 16 μm). This was then subtracted from the image processed with a mean filter of a larger size of 83 × 83 × 83 μm.

To reduce noise outside the organ, we set a threshold at 1.5 times the global average of all images in a sample. After setting this threshold, we refined the mask to better fit the brain sample area. This refinement was achieved through a process known as erosion, which we applied iteratively ten times. Erosion is a mathematical morphology operation that systematically reduces the boundary of regions in an image.^32^ By applying this operation iteratively, we enhanced the specificity of our mask to the brain area, minimizing inclusion of signals from outside the brain. By applying this mask to the Gaussian-mean difference image, we were able to isolate the signal exclusively to the area inside the brain. To isolate tau deposition spots more effectively, we set a threshold for the image at twice the raw intensity value of areas outside the brain sample. Through this thresholding, multiple distinct tau deposition spots were identified within the brain sample area. To systematically analyse these spots, we employed a technique known as connected component labelling. This graph-theoretical method is used to identify and label connected regions (or ‘components’) within a binary image, where a connection is defined by adjacency of pixels.^33^ Each identified tau deposition spot, now considered a ‘connected component’, was given a unique label. This allows for the isolation and individual analysis of each tau deposition spot within the brain, facilitating detailed study of their spatial distribution, intensity and volume. For each tau deposition ROI, we calculated and recorded the centroids, the total intensity of the Gaussian-mean difference, and the volume of the ROI. All these details were saved in a CSV format.

#### CUBIC-cloud registration and anatomical region exclusion

The point cloud data representing tau ROI centroids from the mouse brain were uploaded to the CUBIC-Cloud software, along with the downsampled SYTOX-G images at a resolution of 50 × 50 × 50 μm. Within the CUBIC-Cloud platform, automatic registration was conducted to align these datasets with the CUBIC-Atlas (Supplementary Figs. 4 and 5). For this analysis, data elements corresponding to the ventricular system and fibre tracts were specifically omitted.

### Evaluation of tau detection accuracy

Using anatomical knowledge, we selected specific ROIs with dimensions of 423 × 423 × 423 μm in phospho-Tau (AT8) channel images from three 18-month-old rTKhomo mice, focusing on regions such as anterior cingulate area (ACA), piriform area (PIR), ectorhinal area (ECT), entorhinal cortex (ENT) and CA1. Two experts independently marked pixels in these images they identified as AT8-positive on ImageJ, which we considered as the ground truth. We calculated the *F*-scores by comparing the automated extraction outcomes with the annotations from each expert. Finally, we averaged the two *F*-scores to obtain a unified score for each region in every mouse.

### Workflow of tau gradient consensus analysis

**Figure 2:**
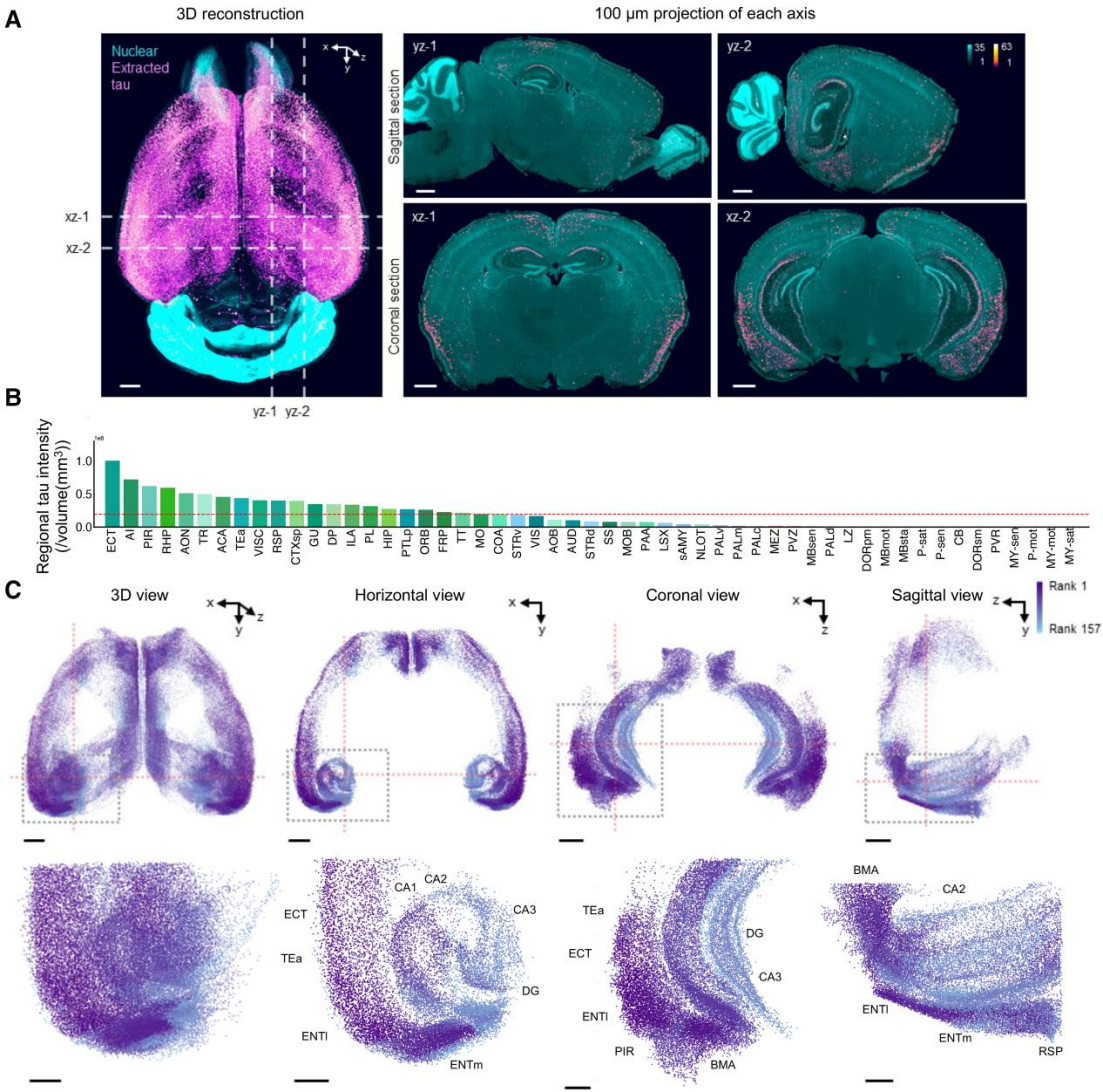
**Whole-brain analysis of tau plaque pathology in rTKhomo mice.** (**A**) A 3D reconstruction and 100 μm projections of both nuclear signal (SYTOX-G) and digitally extracted tau signal (phospho-Tau (AT8)) from coronal and sagittal views of an 18-month-old rTKhomo (Tau+/+, tTA+/−) mouse. The extracted tau signal represents the analysed tau extracted region of interest (ROI), distinguishing it from the background intensity. The data were reconstructed using Imaris software. Scale: 1 mm. (**B**) Integrated tau signal intensity per regional volume across 53 medium-sized subregions, as defined in a previous report.^40^ Integrated tau signal intensity per regional volume was calculated by summing the intensity of tau deposit spots within each anatomical region and normalizing this sum by the total volume of the region (expressed in/mm³). This process was facilitated by the integration of the CCFv3 Allen Brain Atlas^39^ annotations and volume metrics through the use of CUBIC-Cloud.^67^ The presented value is an average derived from data obtained from three 18-month-old rTKhomo mice. The dotted red line represents the mean density value among the 53 regions. (**C**) 3D representation and its horizontal, coronal and sagittal views of the spatial tau plot on atlas space from an 18-month-old rTKhomo mouse. This includes 157 subregions within the medium-sized regions that have a tau density above the mean. Each tau plot represents the centroid position of a detected tau ROI label, with the colour indicating the regional tau density rank in descending order, as shown in the colour bar. The upper three sectional views on the right were created using 3 mm projections from each axis in the 3D whole-brain view. The 3D ROI, outlined by dotted boxes and displayed below in 3D and its horizontal, coronal and sagittal views, comprises 47 regions. The lower three sectional views on the right were created using 3 mm projections from each axis in 3D view. Upper scale: 2 mm. Lower scale: 1 mm.

**Figure 3:**
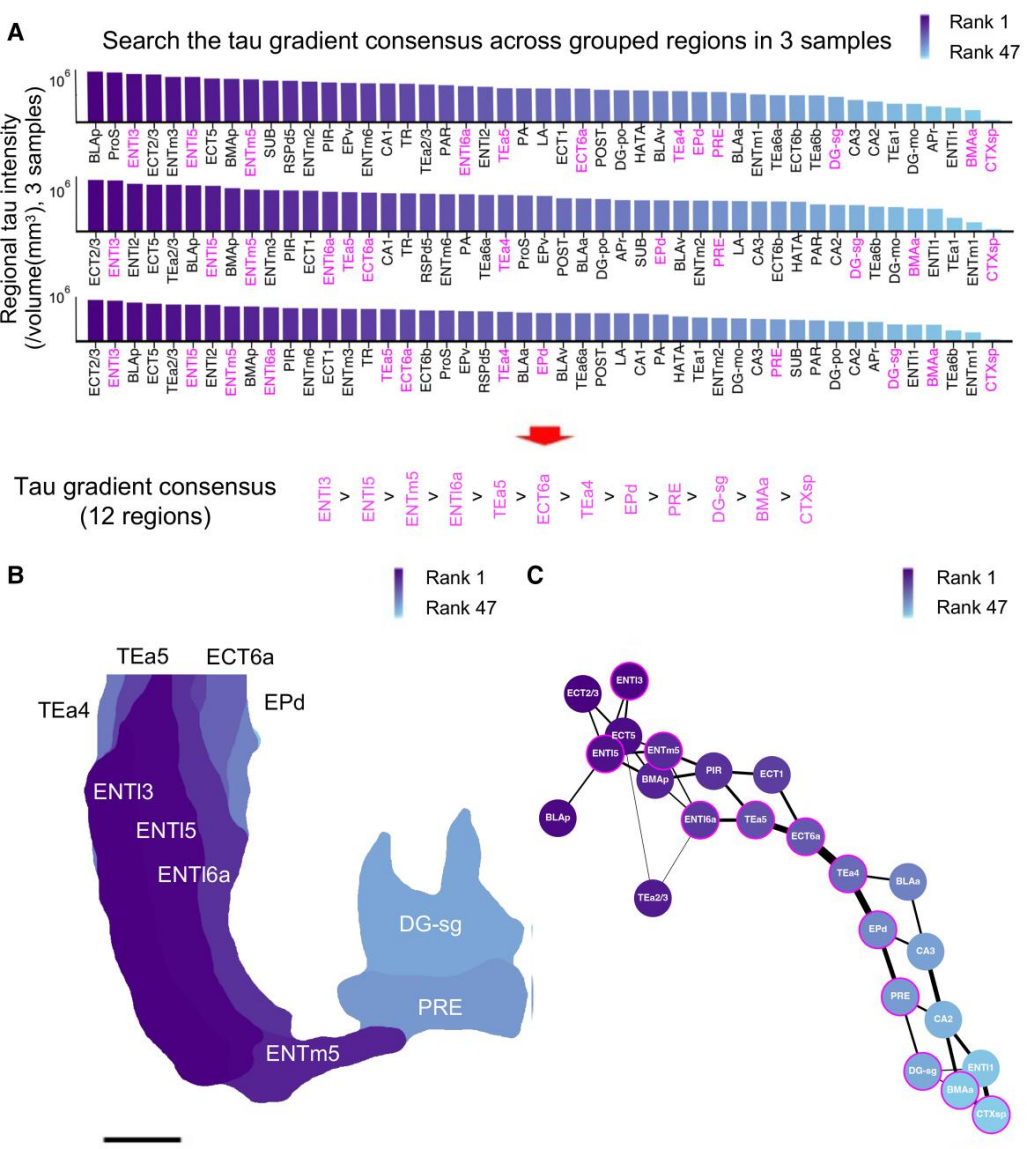
**Tau gradient consensus analysis of tau pathology in rTKhomo mice.** (**A**) A scheme of searching tau gradient consensus across grouped regions in the data from the 47 regions within the 3D ROI determined in Fig. 2C from three 18-month-old rTKhomo mice. Regional tau intensity was calculated by taking the sum of total intensity, dividing it by the volume of each region, and then representing the resulting value in log10 scale. The bars are arranged in descending order based on regional tau intensity. This figure illustrates a representative pattern of regions in a conserved order of tau density, with a maximum sequence length of 12 regions, highlighted in magenta. Consensus refers to the common pattern or sequence observed in the tau density gradient across different mice. (**B**) A representative 3000 μm projection of regional tau density alongside a typical 12-region-sequence tau gradient consensus as highlighted in Fig. 3A. The areas of the regions have been recoloured according to their rank among the 47 regions in descending order. Scale: 1 mm. (**C**) A network graph illustrating the 12-region-sequence tau gradient consensus. Edge weights were initially determined by adding 1 to the adjacency values among the 256 patterns, and subsequently normalized so that the total sum of all weights equals 250. Colours of the nodes correspond to the rank of the mean regional tau density in descending order. Nodes encircled by a magenta line represent the typical 12-region-sequence tau gradient consensus, as highlighted in Fig. 3A.

Tau gradient analysis was introduced for investigating whether there is a consistent trend across multiple samples in the tau deposition gradient, as observed in the representative data of Fig. 2C. Specifically, as depicted in Fig. 3A, our aim was to identify grouped regions where the order of tau intensity was preserved across three samples from 18-month-old rTKhomo mice.

Our study looked closely at 47 regions shown in the bottom parts of Fig. 3A. Searching through all possible orders of these 47 regions would take a lot of computer power. So, we used a simpler method called dynamic programming. It functions like this: if we find a pattern of a certain length, say ‘*n*’, that shows the sequence length of a consistent tau gradient in all three samples, we can assume that this pattern came from a shorter pattern of sequence length ‘*n*−1’. So, by first finding the shortest patterns, we can then build longer ones by adding one region at a time. We began with patterns of just two regions in sequence length and kept adding regions until the patterns no longer showed a consistent tau gradient. The longest pattern we found by this method had a length of 12 regions.

While there are shorter sequences than the longest tau gradient consensus ones, many of these shorter sequences often appear within the longest one. To reveal out unique patterns, we specifically searched for sequences that were not exactly the same as the longer ones, even if portions of them matched. To do this, we began with the longest sequence at 12 and worked our way down in descending order. This method ensured that we were always choosing sequences distinct from longer ones, but we still kept those with some overlapping sections. The results of analysed unique patterns were shown as network graphs at each sequence length in Supplementary Fig. 6B, C.

Considering the statistical likelihood of shorter sequences occurring purely by chance in randomized datasets, we established an empirical significance threshold using the false discovery rate approach. Out of 47 regions from three real data samples were reordered and 10 000 random permutations were performed. A sequence length of 8 was designated as our significance threshold, as fewer than 5% (specifically, 0.0269 at length 8) of these permutations resulted in sequences of this length or greater (Supplementary Fig. 6A). Hence, in our real data, sequences of length 8 or above are considered significant.

Network graphs, as shown in Fig. 3C and Supplementary Fig. 6C, were generated for each sequence length, ranging from 8 to 12, to represent consensus on the tau gradient. The graphs were constructed using the NetworkX library in Python. Nodes were added based on given regions, and edges were placed between adjacent regions. Nodes were positioned using the nx.spring_layout method, employing the force-based algorithm by Fruchterman and Reingold. Edge weights were initially determined by increasing adjacency values among patterns of the same regional length. These weights were later normalized, ensuring that their total sum equaled 250 to preserve visual clarity, as shorter sequences inherently have a greater number of potential combinations and could lead to enormously thicker edges.

### Radiosynthesis

Radiolabelling of PM-PBB3 was performed by the synthetic pathway described previously.^34^ Briefly, tosylate precursor of PM-PBB3 was reacted with ^18^F-fluoride in the presence of dimethyl sulfoxide, K_2_CO_3_ and K222 at 110°C for 15 min. After cooling the reaction vessel to 90°C, hydrochloric acid was added to the mixture and maintained for 10 min to remove the protecting groups. Sodium acetate was then added to the reaction vessel, and the radioactive mixture was transferred into a reservoir for high-performance liquid chromatography (HPLC) purification using a Waters Atlantis prep T3 column (10 × 150 mm; mobile phase, CH_3_CN/50 mM AcONH_4_ = 4/6; flow rate, 5 mL/min). The fraction corresponding to [^18^F]PM-PBB3 was collected in a flask containing 25% ascorbic acid solution and Tween 80 and was evaporated to dryness under a vacuum. The residue was dissolved in 17 mL of saline (pH 7.4) to obtain [^18^F]PM-PBB3 as an injectable solution. The final formulated product was radiochemically pure (≧95%) as detected by analytic HPLC (Waters Atlantis prep T3 column, 4.6 × 150 mm; mobile phase, CH_3_CN/50 mM AcONH_4_ = 4/6; flow rate, 1 mL/min).

### MR and PET imaging

MR imaging and PET scans were conducted as described in Kimura *et al*.^28^ to assess whether tau deposition could be detected in our novel model mice using *in vivo* imaging. MRI was conducted on mice under isoflurane anaesthesia using a facial mask (3% v/v for induction, followed by 1.5–2% for maintenance), with body temperature maintained at 36.5°C–37.0°C via a heating pad. T2-weighted (T2W) magnetic resonance (MR) images were acquired with a 7.0 T horizontal MRI scanner (Magnet: Kobelco and JASTEC, Japan; Console: Bruker Biospin, Germany) with a volume coil for transmission and a 2-ch phased array surface coil for reception (Bruker Biospin). The imaging parameters were: TR/effective TE = 4200/36 ms, Fat-Sup = on, NA = 8, RARE factor = 8, FOV = 25.6 × 14.5 × 14 mm, matrix = 256 × 256 × 28, voxel size = 0.1 × 0.057 × 0.5 mm, scan time = 13 min 26 s.

PET scans were conducted using a microPET Focus 220 animal PET scanner (Siemens Medical Solutions, USA). Mice were kept under isoflurane anaesthesia, and their body temperature was maintained throughout the PET experiments as in the MRI experiments. A 30G needle connected to a polyethylene tube (SP-10, Natsume Seisakusho, Co. Ltd., Tokyo, Japan) was inserted into the tail vein of the mice, which was then placed on the PET acquisition bed. Immediately after the intravenous injection of [^18^F]PM-PBB3, list-mode emission data with an energy window of 350–750 keV were acquired for 60 min. Injected radioactivity and molar activity at the time of [^18^F]PM-PBB3 injection were 17.20 ± 0.96 MBq and 155.3 or 168.4 GBq/μmol. Emission data were sorted into 3D sinograms (20 frames; 4 × 1 min, 8 × 2 min and 8 × 5 min) and then Fourier-rebinned into 2D sinograms. Images were reconstructed using filtered-back projection with a Hanning filter at a cutoff frequency of 0.5 mm^−1^. No attenuation and scatter corrections were applied. The reconstructed images had a matrix size of 128 × 128 × 95 with a voxel size of 0.38 × 0.38 × 0.80 mm.

### Quantification of [^18^F]PM-PBB3 binding

PET images were co-registered with the individual MR images, and volume of interests (VOIs) of the hippocampus and cerebellum were placed to obtain regional time-activity curves (TACs). VOIs were created using MR images according to a previous report.^35^ The average radioactive signals (kBq/cc) in each of the VOIs were then normalized by the injected radioactivity and body weight and were expressed as standardized uptake values (SUVs). Then, the standardized uptake value ratio (SUVR) of [^18^F]PM-PBB3 was calculated by dividing the SUV of the target region by the SUV of the cerebellum, as the cerebellum was devoid of tau aggregates in this mouse model. All PET imaging and kinetic analyses described above were performed using PMOD software (Ver. 4.206, PMOD Technologies LLC, Switzerland).

### Image quantification of 2D staining

Averaged areas (mm^2^) of dentate granule cell layers in 2-month-old mice (genotype: tTA or control, strain: C57BL/6J or FVB/129) were defined by DAPI positive signals in hippocampi from three serial sagittal sections of each mouse (three mice per group) by BZ-X Analyzer (Keyence). For image quantification of IBA1 or GFAP positive signal in the DG of 2-month-old mice (genotype: tTA or control, strain: C57BL/6J or FVB/129), the area of the IBA1- or GFAP-positive signal was extracted and quantified by BZ-X Analyzer. The IBA1 or GFAP positive signal was displayed as the signal ratio (%) to the total area (0.52–0.84 mm^2^ or 0.30–0.78 mm^2^ field of DG, respectively). To examine whether tau-induced neuronal loss had occurred in rTKhomo mice, the NeuN- or Hoechst33342-labelled hippocampal area was measured for fluorescence signals by BZ-X Analyzer. Thickness of the cell layer was quantitated with vertical lines drawn from the outside of each point (CA1; seven points, CA2 + CA3; 5 points, DG: 8 points). Cell layer thickness of each region was calculated from the average of each point (control: five mice, KI: seven mice). For image quantifications of IBA1and GFAP positive signals in hippocampal areas of rTKhomo mice, the positive signals were extracted and quantified by BZ-X Analyzer. Immuno-positive levels were displayed as the signal ratio (%) to the total area (0.11–0.23 mm^2^ field of hippocampal areas). Quantification of tau pathology was examined by counting for hyperphosphorylated tau-specific antibody AT8-positive neuronal inclusions in sagittal brain sections from 3 to 18-month-old rTKhomo mice. Measurements were manually performed on three serial sagittal sections of each mouse by three experienced investigators. For image quantifications of IBA1 positive cell size, the positive signals were extracted and quantified by BZ-X Analyzer. Using the same condition thresholds in each group, ROIs recognizing cells were created and their areas were calculated. For image quantifications of AT8 and P2RY12 positive signals in hippocampal subregions of rTKhomo mice, the positive signals were extracted and quantified by ImageJ. Images were converted to 8-bit and quantified with respective thresholds (AT8:40–255, P2RY12:30–255). ROI was based on the Allen Mouse Brain Atlas, mouse.brain-map.org and atlas.brain-map.org. For image quantifications of AT8, IBA1 and GFAP positive signals in hippocampal areas of human postmortem brain samples, the positive signals were extracted and quantified by BZ-X Analyzer. Immuno-positive levels were displayed as the signal ratio (%) to the total area (0.42 mm^2^ field of hippocampal areas).

### Statistical analysis

Statistical analysis was conducted using PRISM7 or 9 (GraphPad Software Inc., La Jolla, CA). For comparison of the two groups, data were analysed by Unpaired *t*-test or Mann–Whitney test. For multiple comparisons between groups, one-way ANOVA followed by Tukey’s multiple comparisons test was performed. Statistical significance was determined by *P*-value < 0.05.

## Results

### Generation and pathological tau accumulation in Rosa26 knock-in mice

We employed the locus-specific knock-in method and the Tet-off system to establish a novel mouse model of tauopathy, aimed at minimizing artifacts and achieving controlled overexpression of human tau. To accomplish locus-specific and a single copy transgene expression, P301L mutated human 1N4R tau cDNA with the Tet-responsive *P*_tight_ promoter was translocated into the *Rosa26* locus (Supplementary Fig. 1A). Subsequently, we crossed Rosa26 knock-in mice with tTA tg mice (Supplementary Fig. 1B). Hereafter, we refer to mice with homozygous Rosa26 knock-in Tau expression (Tau+/+, tTA+/−) as rTKhomo, and they exhibited twice the level of human tau protein expression compared with their heterozygous counterparts (Tau+/−, tTA+/−), donated as rTKhetero (Supplementary Fig. 1C). To quantify the human tau protein level, we compared the band intensity of human 1N4R tau to that of mouse 0N4R tau. The human tau protein, as detected by Tau5 or Tau46 antibody, displayed a 7-fold or 5-fold increase relative to mouse 0N4R tau, respectively (Supplementary Fig. 1D, E). Notably, there was no significant difference in the level of mouse endogenous tau protein between rTKhomo mice and tau responder mice (Supplementary Fig. 1F). Our biochemical and immunohistochemical analyses corroborated the expression of human tau in rTKhetero mice, whereas human tau protein remained unidentifiable in tau responder mice, signifying the lack of leaky expression of inserted protein, as observed in the previous study (Fig. 1A, B).^36^ Consistent with previous findings,^16^ the reduction in thickness of the dentate granule cell layer in FVB/129 mixed background strains was significantly ameliorated when the tTA tg mouse was backcrossed onto a C57BL/6J background (Fig. 1C, D). In the DG of FVB/129 mixed background strains, there was a significant elevation in IBA1-positive microglia and GFAP-positive astrocytes in comparison to the B6 strain (Fig. 1C, E and F). This suggests that tTA-induced glial activation occurs within the dentate region of mice with a mixed FVB/129 background. On the other hand, in rTKhomo mice, tau pathology was found in the forebrain region at 18 months of age (Fig. 1G, I). Immunostaining with phosphorylation-dependent tau antibody AT8, conformation-dependent tau antibody MC1 and pan-tau antibody HT7 of the hippocampus in sagittal sections revealed the deposition of AT8− and MC1-positive tau aggregates, unlike rTg4510, specifically in CA1 (Fig. 1G–J), despite the absence of regional differences in HT7-positive tau expression (Supplementary Fig. 1G–N). Notably, it was demonstrated that MC1-positive mossy fibres remained intact in rTKhomo mice compared with rTg4510 (Fig. 1I, J).

Next, we analysed the stability of human tau expression and the progression of age-dependent tau pathology at each month of age. As previously reported,^29^ brain extracts from 3- to 18-month-old rTKhomo mice were fractionated into TBS-extractable and sarkosyl-insoluble fractions. These fractions were analysed by western blotting using tau antibodies. Blotting with phosphorylation-independent tau antibodies (e.g. Tau12 and Tau46) showed constant levels of tau protein in the TBS-extractable fraction (Supplementary Fig. 2A–C). On the other hand, blotting with phosphorylation site-specific tau antibodies (e.g. AT8 and PHF1) showed an intense band that migrated to 68 kDa (indicated by arrow in Supplementary Fig. 2A) in the same fraction from 15-month-old (Supplementary Fig. 2A, D and E). This hyperphosphorylated 68 kDa tau was clearly observed in blotting with sarkosyl-insoluble fractions. The 68 kDa tau appeared at 12- to 15-month-old and increased with age (Supplementary Fig. 2F–J). Biochemical observations of hyperphosphorylated tau in both TBS-extractable and sarkosyl-insoluble fractions from aged mice revealed that accumulations of pathological tau species appeared as early as 12 months of age.

Considering the reported sex-specific alterations in neuropathology in various tauopathy mouse models,^37,38^ we investigated sex differences in human tau expression and pathological tau formation in rTKhomo mice. Results revealed no discernible difference between males and females in the TBS-soluble fraction, both in 15-month-old and over 18-month-old rTKhomo mice (Supplementary Fig. 3A–E). Similarly, in the sarkosyl-insoluble fraction, there were no significant differences between males and females in both 15-month-old and over 18-month-old rTKhomo mice, although the variability of hyperphosphorylated 68 kDa tau was higher in 15-month-old mice compared with that in over 18-month-old mice (Supplementary Fig. 3F–J). Given the onset of pathological tau species accumulation from 12 months of age, a broader spectrum of biochemical analyses will be necessary to validate sex-specific alterations in pathological progression in future studies.

### Whole brain analysis of pathological tau accumulation

To investigate localization of the tau pathology in this novel model mouse, whole organ 3D immunohistochemical examination with AT8 antibody in addition to SYTOX-Green nucleic acid staining was performed on 18-month-old rTKhomo mice. AT8 was used in this study because of its good performance in staining phosphorylated paired helical filament tau. AT8 immunostaining revealed the hyperphosphorylated intraneuronal tau inclusions distributed in the forebrain region (Fig. 2A). In Fig. 2B, integrated tau signal intensity per regional volume across 53 medium-sized subregions were aligned from higher to lower levels (left to right). This metric was derived by summing the signal intensity of tau deposits in each anatomical area, then normalized by the region's total volume, leveraging annotations and volume metrics from the CCFv3 Allen Brain Atlas.^39^ The displayed values represent averages from analyses of three 18-month-old rTKhomo mice. Brain subregions were divided into 53 medium-sized regions according to a previous report.^40^ Higher levels of AT8-positive tau signals were observed in ECT and the agranular insular area (AI) of the isocortex, PIR of the olfactory area and the retrohippocampal region (RHP) of the Hippocampal formation (Fig. 2B). This trend was also validated using the ratio of integrated AT8-positive tau volume per region volume (Supplementary Fig. 4A). For more detailed analysis of AT8-positive tau distribution, integrated tau signal intensity per regional volume analysis using CUBIC-Cloud software (https://cubic-cloud.com) was examined (Supplementary Figs. 4 and 5). In the isocortex, the dorsal part of the anterior cingulate area (ACAd), agranular insular areas (AId, AIp and AIv), perirhinal area (PERI), and ECT showed accumulation of AT8-postive tau in layers 2/3 and 5. AT8-postive tau was also distributed in the layer 2/3 of anteromedial visual area (VISam), postrhinal visual area (VISpor) and anterior visual area (VISa) (Supplementary Fig. 4D). Moreover, higher levels of AT8-positive tau signals were observed in the lateral entorhinal area (ENTl) and the medial entorhinal area (ENTm) of the RHP and the anterior basolateral amygdala nucleus BLAp and the posterior basomedial amygdalar nucleus (BMAp) of the cortical subplate (CTXsp) (Supplementary Figs. 4E and 5). Figure 2C showed the density of the 157 subregions within the medium-sized regions above the mean signals. As shown in the high-magnified images (Fig. 2C), AT8-postive tau signals were remarkably increased in ENTI, ENTm and BMA, indicating further confirmation of the unique distribution pattern of tau pathology in rTKhomo mouse brains.

Next, tau gradient analysis was introduced to investigate whether there was a consistent trend across multiple samples in the tau deposition gradient (Fig. 3A–C, see Materials and Methods). Our primary aim was to identify grouped regions where the order of tau intensity was preserved across three individuals of 18-month-old rTKhomo mice. As a result, 12 regions [i.e. ENTI3, ENTI5, ENTm5, ENTI6a, temporal association area 5 (TEa5), ECT6a, TEa4, dorsal endopiriform nucleus (EPd), (presubiculum) PRE, DG-sg, BMAa and CTXsp] with identical order of AT8-postive tau signals in three independent mice were observed (Fig. 3A and B, Supplementary Fig. 6). The tau network diagram of the 12 regions and related brain regions (Fig. 3C) seems to indicate neural connectivity in the hippocampal formation. To clarify a primary region of pathological tau accumulation, younger rTKhomo mice around 12 months of age were examined. At this age, AT8-positive intra-neuronal tau deposition was observed around the ENT (Supplementary Fig. 7) but not in the hippocampal region (HIP). Therefore, pathological tau could be deposited in the ENT and spread to the hippocampal formation with a conserved sequence.

### *In vivo* [^18^F]PM-PBB3 PET imaging for tangle-formed tau

**Figure 4:**
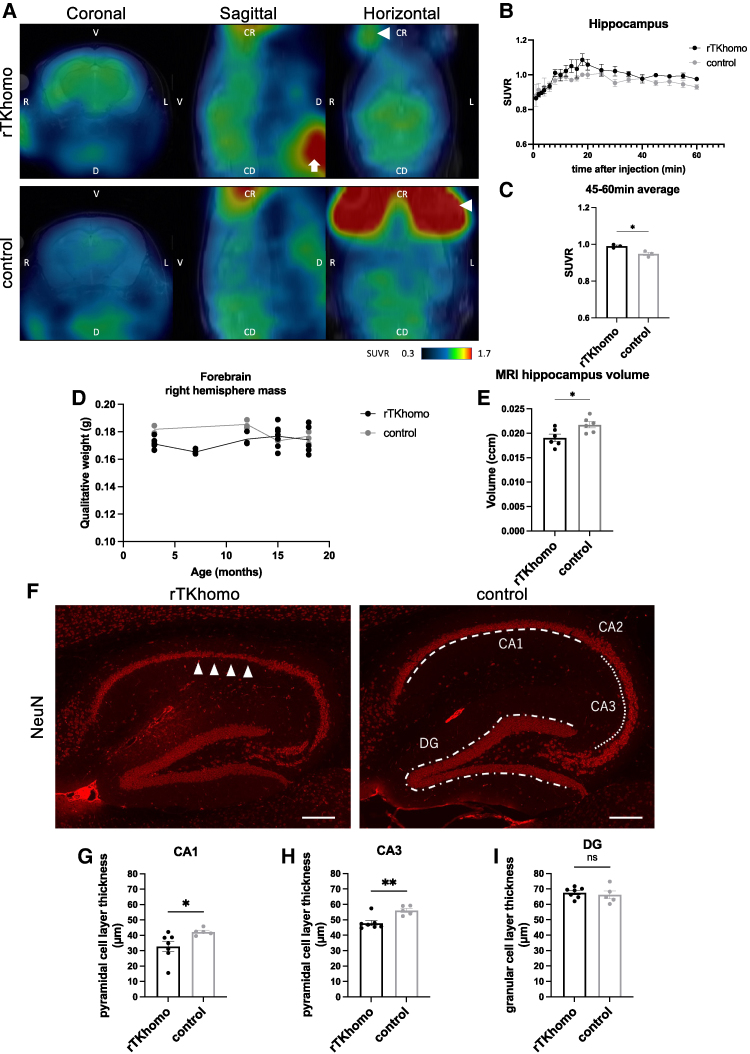
***In vivo* imaging for tau tangle formation using [^18^F]PM-PBB3 PET and neurodegeneration in hippocampus.** (**A**) [^18^F]PM-PBB3 the standardized uptake value ratio (SUVR) images (cerebellar signal as reference) in 18-month-old rTKhomo (Tau+/+, tTA+/−) and control (Tau+/+, tTA−/−) mice. These images were generated by averaging dynamic data 14–60 min after tracer injection and superimposed on individual MRI T2-weighted images. L, left; R, right; V, ventral; D, dorsal; CR, cranial; CD, caudal. Arrow and arrow heads were signals from non-specific accumulation of [^18^F]PM-PBB3 to submandibular gland (white arrow) and Harderian gland (white arrowheads). (**B**) Time courses of SUVR in hippocampus of rTKhomo and control mice. Data are presented as mean ± SEM. (**C**) Comparisons of regional SUVR values 45–60 min after tracer injection between two genotypes (rTKhomo, *n* = 3; control, *n* = 3). Values are mean ± SEM. Unpaired *t* test was performed (*P < 0.05). (**D**) Forebrain right hemispheres were dissected from 3−, 7−, 12−, 15− and 18-month-old mice for western blotting and weighed. There was no significant difference between the two groups. (**E**) Volumetric analysis of hippocampus of 18-month-old rTKhomo and control mice by MRI T2-weighted imaging (rTKhomo, *n* = 6; control, *n* = 6). Values are mean ± SEM. Unpaired *t* test was performed (*P < 0.05). (**F**) NeuN immunoreactivities in hippocampus from 18-month-old rTKhomo and 15–18-month-old control mice. Arrowheads indicate thinner CA1 pyramidal cell layer observed in rTKhomo mice. Dotted lines indicate pyramidal cell layers of CA1, CA2, CA3 and dentate granular cell layer (DG). Scale bars = 200 µm. (**G–I**) Thicknesses of CA1 (**G**) and CA3 (**H**) pyramidal cell layers and DG (**I**) in 18-month-old rTKhomo (male, *n* = 1; female, *n* = 6) and 15–18-month-old control (male, *n* = 4; female, *n* = 1) mice. Thickness values are mean ± SEM. Unpaired *t* test was performed (**P < 0.01, *P < 0.05).

Since AT8-positive intracellular tau accumulation was detected by whole brain analysis, we expected that tau pathology in rTKhomo mice could be visualized by tau PET imaging. According to the previous report,^34^ PET using the ^18^F labelled β-sheet ligand PM-PBB3 can detect matured Tau tangles in living mice. Thus, we examined [^18^F]PM-PBB3 PET imaging of rTKhomo mice to determine whether tau pathology can be detected *in vivo*. For the VOI analysis, the setting of the VOI area and the selection of the reference area were based on our previous reports.^28,35^ Figure 4A shows representative [^18^F]PM-PBB3 PET images of the rTKhomo and control mouse brains at 18 months of age. The radiotracer SUVR, regional radioactivity of [^18^F]PM-PBB3 in the hippocampus area, was quantified by reference region (SUVR = hippocampus-SUV/cerebellum-SUV) (SUV = radioactivity in VOI (kBq/cc)/[dose radioactivity (kBq)/body weight (g)]) and reached a plateau around 14 min after injection (Fig. 4B). The SUVR values of the 18-month-old rTKhomo mouse hippocampi corresponded to the values of 3- to 4-month-old rTg4510 mice reported in a previous study.^28^ The SUVR values at 14–60 min in rTKhomo mice were slightly but significantly higher than those in control mice (Fig. 4C). These data suggest that the aged rTKhomo mice developed mature tau pathology that can be visualized by Tau PET imaging.

### Neurodegeneration in the hippocampal region of rTKhomo mice

Because AT8 and MC1 immunostaining revealed intraneuronal hyperphosphorylated tau inclusions in the hippocampal CA1 (Fig. 1G–J), we examined whether neuronal loss occurred in this area of rTKhomo mice. There was no age-dependent and significant difference in wet weights of the right hemisphere, excluding the cerebellum and olfactory bulb, between the rTKhomo and control mice (Fig. 4D). When brain volume was evaluated by T2W MRI images, a slight decrease in hippocampal volume in rTKhomo mice was observed (Fig. 4E). For further evaluation, neuronal cell volumes of the hippocampal cell layer were measured by NeuN staining, referring to the method of a previous report.^16^ As results, thickness of the NeuN-positive hippocampal CA1 and CA3 pyramidal cell layers of 18-month-old rTKhomo mice was significantly less than that of control mice, but there was no significant difference in dentate granular cell layers between the rTKhomo and control mice (Fig. 4F–I). These quantitative data suggest that pathological tau formation eventually induces neuronal cell loss in a brain regional-specific manner. Unlike rTg4510 and others,^7,8,10^ there was no significant loss of brain mass. This could be an advantage for whole brain 3D analysis because brain atrophy and/or deformation may affect voxel-based analysis due to the difficulty of brain normalization.

### Pathological tau-associated glial phenotypic change

**Figure 5:**
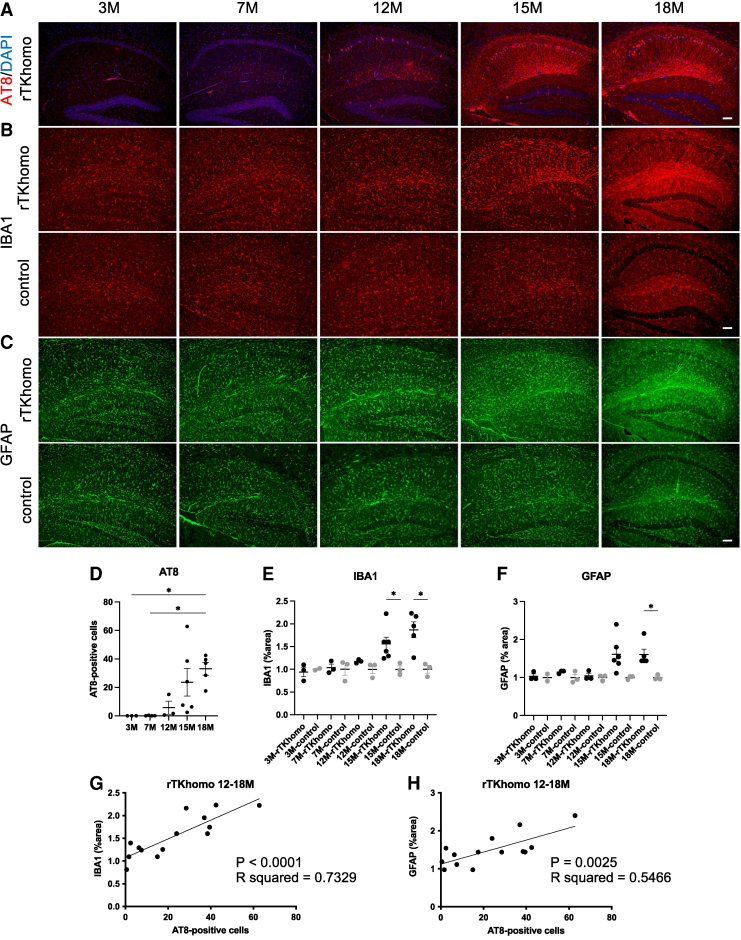
**Age-dependent glial phenotypic changes in hippocampi of rTKhomo and control mice.** (**A**) Representative AT8 immunofluorescence labelling images in hippocampi of 3−, 7−, 12−, 15−, 18-month-old rTKhomo (tau+/+, tTA+/−) mice. (**B**) Representative IBA1 immunofluorescence labelling images in hippocampus areas of 3−, 7−, 12−, 15−, 18-month-old rTKhomo and control (tau+/+, tTA−/−) mice. (**C**) Representative GFAP immunofluorescence labelling images in hippocampus areas of 3−, 7−, 12−, 15−, 18-month-old rTKhomo and control mice. (A–C) Scale bars = 100 µm. (**D**) Semi-quantification of AT8-positive cell numbers in hippocampus areas of rTKhomo mice. Tukey’s multiple comparisons test was performed (*P < 0.05). Values are mean ± SEM. (**E**) Semi-quantification of IBA1 signals in hippocampus of rTKhomo and control mice. Values are mean ± SEM with respect to levels measured from samples of age-matched control mice; those levels were scaled to a value of one. (**F**) Semi-quantification of GFAP signals in hippocampus of rTKhomo and control mice. Values are mean ± SEM with respect to levels measured from samples of age-matched control mice; those levels were scaled to a value of one. 3− (male, *n* = 3), 7− (male, *n* = 1 (AT8 = 2); female, *n* = 2), 12− (male, *n* = 1; female, *n* = 2), 15− (male, *n* = 3; female, *n* = 3), 18-month-old (female, *n* = 5) rTKhomo and 3− (male, *n* = 2), 7− (male, *n* = 3), 12− (female, *n* = 3), 15-month-old (male, *n* = 3), 18-month-old (male, *n* = 1; female, *n* = 2) control mice were examined. Since the values for rTKhomo mice are shown as a ratio to the age-matched control group, unpaired *t* test was performed between groups of the same age (*P < 0.05). (**G** and **H**) Scatterplots of IBA1 (**G**) and GFAP (**H**) immunoreactivities in hippocampi for AT8-positive cell numbers of rTKhomo mice at 12–18 months of age. Pearson correlation coefficient showed significant correlations (G, *P* < 0.0001; *R*^2^ = 0.7329, H, *P* = 0.0025; *R*^2^ = 0.5466).

As significant features of rTKhomo mice, we observed that neurons bearing AT8-positive tau were accumulated in hippocampal CA1 (Fig. 1G), and that neurodegeneration occurred in the CA1 pyramidal cell layer (Fig. 4G). In addition, since rTKhomo mice were generated under a C57BL/6J strain background, thinning of the dentate granule cell layer and glial activation, which were observed in an FVB/129 mixed strain background, were absent in this model (Fig. 1C–F). In addition, tTA toxicity was reported to be absent until 12 months of age.^16^ Therefore, the rTKhomo mouse line is potentially more suitable for analyzing the tau-induced microglial response than other models such as rTg4510 and rT2.^7,10^ Here, we examined the immunohistochemistry on sagittal sections of rTKhomo mice from 3- to 18-month-old to determine whether glial inflammatory response was dependent on tau pathology. AT8-positive cell processes in the CA1 region and cellular inclusions appeared at 12 months of age and then increased with age (Fig. 5A, D). The same individuals showed an age-dependent increase of IBA1-positive microglia and GFAP-positive astrocyte from 7 months of age compared with age-matched control mice (Fig. 5B, C, E and F). While there was noticeable individual variability in both IBA1 and GFAP signals at 15–18 months of age was evident, statistical analysis demonstrated a significant difference between rTKhomo and control mice in the IBA1 signal at 15 and 18 months of age, and in the GFAP signal at 18 months of age (Fig. 5E, F). To further confirm the significant relationships between tau pathology and glial proliferation, correlations between immunofluorescent signals of glial markers (e.g. IBA1 and GFAP) and AT8-positive neuronal numbers in individual mice at 12–18 months of age were examined. There were significant correlations in all comparisons, suggesting evidence of tau pathology-dependent glial activation (Fig. 5G, H). Tau pathology-dependent glial activation was further confirmed in the hippocampal section of human SD-NFT,^25,26^ which is characterized by accumulations of tau fibrils but lacks marked amyloid-β (Aβ) deposits and non-demented subjects (Supplementary Fig. 8).

**Figure 6:**
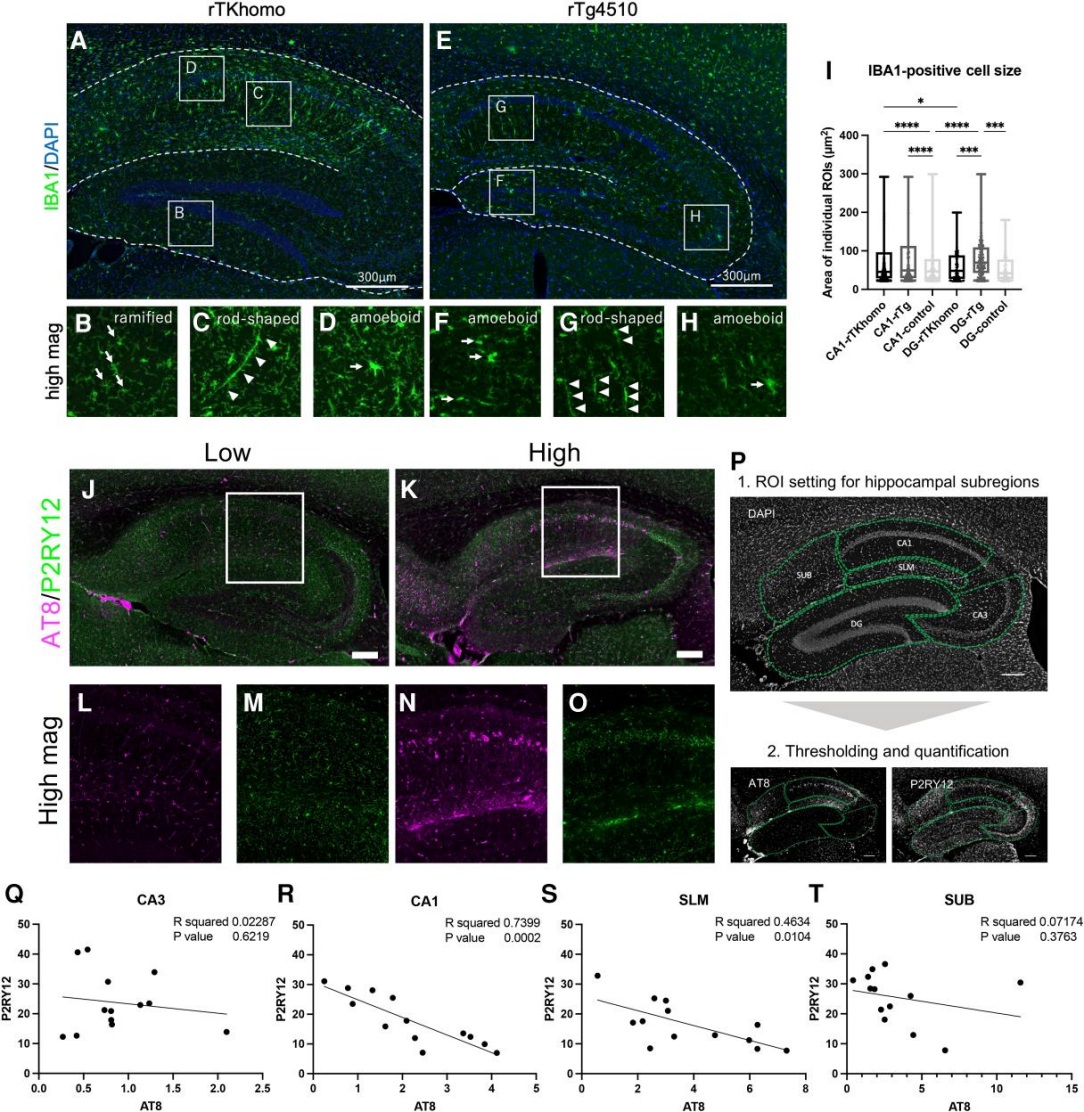
**Morphological diversity and spatially tau-dependent response of microglia in the hippocampi of rTKhomo mice.** (**A** and **E**) Brain sections of 18-month-old rTKhomo (tau+/+, tTA+/−) mice and rTg4510 were immune-stained by IBA1 antibody. Scale bar = 300 µm. (**B**) Representative images of ramified microglia in dentate gyrus (DG). Arrows show microglial cell bodies. (**C** and **G**) Rod-shaped microglia in the stratum radiatum of CA1 region. Arrowheads indicate process tracking of rod-shaped microglia. (**D**, **F** and **H**) Amoeboid-like microglia in CA1 pyramidal cell layer of rTKhomo mice, CA3 and DG of rTg4510. Arrow shows microglial cell body. (**I**) Quantification of IBA1-positive microglial cell body size in CA1 and DG. 18-month-old rTKhomo (plots include 5339 cells in CA1 and 884 cells in DG from five females; minimum 1003 cells/animal in CA1 and 133 cells/animal in DG, respectively), 6–7-month-old rTg4510 (plots include 2661 cells in CA1 and 1049 cells in DG from two males and three females; minimum 414 cells/animal in CA1 and minimum 117 cells/animal in DG, respectively), 18-month-old control (plots include 2162 cells in CA1 and 293 cells in DG from one male and two females; minimum 535 cells/animal in CA1 and minimum 86 cells/animal in DG, respectively) mice were examined. Box and whiskers plotted Min to Max, show all points. Tukey’s multiple comparisons test was performed on the mean values of each group. (*P < 0.05, ***P < 0.001, ****P < 0.0001). (**J** and **K**) Representative AT8 and P2RY12 immunofluorescence labelling images in hippocampi of 15− and 18-month-old rTKhomo mice. The number of AT8-positive neurons was low (**J**), high (**K**). Scale bars = 200 µm. (**L–O**) High magnification images of top images, the region of CA1, the regions of the stratum lacunosum-moleculare and DG. (**L** and **N**) AT8 immunofluoresence labeling images. (**M** and **O**) P2RY12 immunofluoresence labeling images. (**P**) Procedure for quantification of AT8 and P2RY12 signals and representative image of region of interest (ROI) for hippocampal AT8-positive and P2RY12 positive signals. Using imageJ software, we set ROIs based on the DAPI signal and quantified the positive area of binarized AT8 and P2RY12 signals. DG, dentate gyrus; SLM, stratum lacunosum-moleculare; SUB, subiculum. Scale bars = 200 µm. (**Q–T**) Scatterplots of P2RY12 signal (% area) in hippocampal regions for AT8 signal (% area) of rTKhomo mice at 15–18 months of age. 15-month-old (male, *n* = 6; female, *n* = 3), 18-month-old (female, *n* = 4). Pearson correlation coefficient showed significant correlations (R, *P* = 0.0002; *R*^2^ = 0.7399, S, *P* = 0.0104; *R*^2^ = 0.4634).

Next, we examined the morphological features of microglia in the hippocampal subregions of tauopathy mouse models. In rTKhomo mice, we observed typical ramified type microglia in DG whereas morphological activation (e.g. rod-shaped, amoeboid)^41,42^ was observed in CA1 (Fig. 6A—D). These morphological differences are likely due to the presence or absence of tau pathology (Fig. 5A). On the other hand, rTg4510 shows morphological activation in the entire hippocampal areas (Fig. 6E–H). Since microglial soma size is one of quantitative parameters for classifying microglial morphological phenotypes (e.g. ramified microglia have smaller soma size, while amoeboid and rod-like microglia have larger soma size),^43^ we conducted cell measurement analysis on IBA1-positive microglia in the hippocampal areas. Our data revealed significantly larger soma size in the hippocampal CA1 region compared with the DG in 18-month-old rTKhomo mice (Fig. 6I). Moreover, the soma size in the DG of 6.5-month-old rTg4510 mice was larger than that in the DG of both rTKhomo and control mice, while no difference in soma size was observed in the DG region between rTKhomo and control mice (Fig. 6I). To reveal more detailed local microglial phenotypic changes that may be a response to tau pathology, we analysed the correlation between tau pathology and homeostatic microglia reduction in the hippocampal subregions. We performed co-immunostaining AT8 and P2RY12 (homeostatic microglial marker) of 15- to 18-month-old rTKhomo mice. Representative images from two independent rTKhomo mice showed different levels of AT8-positive signals in the hippocampal CA1 (low level in Fig. 6J, higher level in Fig. 6K). Higher magnified observations showed that AT8-positive signals appeared to increase in the pyramidal neuronal cell somas and processes in the CA1 pyramidal layer to the stratum lacunosum-moleculare (SLM) region (Fig. 6L, N). In association with the progression of AT8-positive tau distribution, IBA1-positive microglial proliferation was observed in the same hippocampal subregions (Fig. 5B and E). In contrast to IBA1 immunostaining, P2RY12-positive homeostatic microglia appeared to be decreased in hippocampal CA1 along with the increase of AT8-positive tau (Fig. 6M, O). To investigate relationship between homeostatic microglia and tau pathology, ROIs were set in the hippocampal subregions (Fig. 6P), and the correlation between AT8 signal and P2RY12 immunoreactivity was analysed (Fig. 6Q–T). Significant correlation was observed in the CA1 pyramidal layer and SLM (Fig. 6R, S) while there was no significant correlation in the hippocampal CA3 and subiculum (Fig. 6Q, T). These results indicate that our model recapitulates the tau-induced inflammatory response in microglia.

## Discussion

### Pathological features of rTKhomo mice

In this study, we have established a novel murine model of tauopathy characterized by the expression of human P301L mutated tau under the regulation of a Tet-off controlled CaMKII promoter. The stable expression of human tau protein was achieved through the integration of a human 1N4R tau transgene into the *Rosa26* locus. Notably, rTKhomo mice exhibited age-dependent regional-specific accumulation of pathological tau, with discernible AT8-positve tau deposits evident in 18-month-old mice. This model is strongly suggestive of replicating the tau pathogenesis observed in Alzheimer’s disease, given that ENT is a primary site of NFT formation, as per Braak NFT staging.^24^ Furthermore, employing tau PET imaging allowed for the visualization of *in vivo* pathology in aged rTKhomo mice. The successful detection of tracer uptake in pathological areas, as previously reported,^28,34^ implies the presence of mature neurofibrillary pathology. In concert with the progression of tau pathology, a thinning of the hippocampal pyramidal cell layer became apparent in aged rTKhomo mice. Within the same region, microglia and astrocytes exhibited activation, mirroring the features observed in human neurodegeneration.^13,44-47^ Collectively, our present murine model recapitulates crucial aspects of tauopathy, encompassing NFTs, neuronal loss and neuroinflammation while reducing confounding artificial phenotypes resulting from one of the genome editing errors.

### Utility of our strategy and distinct tau pathology in rTKhomo mice

The utilization of human tau variants associated with FTDP-17 mutations offers a viable approach for the creation of a murine model that faithfully recapitulates the pathological characteristics observed in human tauopathy.^3,48^ In this study, we succeeded in inducing overexpression of P301L mutated human tau by targeted insertion in *Rosa26* and driving by Tet-off system. Similar to Rosa26 Knock-in Tau mice, a knock-in mouse (named T2) was previously generated with a single targeted P301L mutated human tau insertion in the downstream of *Col1A1*.^10^ Crossing T2 mice with tTA tg mice produced rT2 mice that express P301L mutated human 0N4R tau 8.5-fold more than endogenous mouse tau. A side-by-side comparison is necessary, although this level of expression exceeded that observed in rTKhetero mice harboring P301L mutant human 1N4R tau (Supplementary Fig. 1D, E). The observed disparity in expression levels may stem from variations in integration sites, although other factors such as Tet-responsive promoters, tau isoforms and strain background could also influence transgene expression. Remarkably, the protein levels of human tau in both lines surpassed those of endogenous mouse tau, with a dependence on copy number. Despite the differences in strain background, both lines shared the same tTA tg mice ((CaMKIIa-tTA)1Mmay mice). This underscores the critical role of the Tet-off system and CaMKIIa promoter-controlled tTA expression in achieving successful overexpression of human tau. However, it is important to acknowledge that the presence of unknown factors affecting the overexpression of human tau cannot be ruled out, given the presence of gene disruption (*Vipr2, Wdr60, Esyt2, D430020J02Rik*, *Ncapg2* and *Ptprn2*) in tTA tg mice. To mitigate these uncertainties, generating tTA overexpressing models devoid of any gene disruption, employing targeted knock-in strategies, and using the Tet-off-drive system can prove to be potent tools for enhancing the expression of a target protein.

Given the regulation of human P301L tau expression through tTA under the CaMKII promoter,^49^ the resulting expression is primarily localized within the forebrain, encompassing the neocortex, hippocampus, amygdala and striatum. The verification of this distribution of human P301L tau protein in rTKhomo mice was confirmed through immunofluorescence staining utilizing the Tau12 antibody, which specifically targets phosphorylation-independent human tau protein (Fig. 1B). To uncover the brain-wide pathological tau distribution, recent advances in whole brain three-dimensional (3D) staining and imaging^50^ had revealed that AT8-positive tau pathology in rTKhomo mice predominantly along the entorhinal cortex-hippocampal projection extended from ENT to various brain regions, including TEa, ECT, PRE, subiculum (SUB), DG and anmon’s horn (CA1, CA2 and CA3) (Fig. 2). These deposition sites were similar to the corticohippocampal circuit classically identified as functional connections^51^ and contained SUB and ENT connections important for spatial working memory identified in a previous report.^52^ Notably, within the hippocampus, the highest AT8 signal density is observed in CA1 (Supplementary Figs. 4E, 5A and 6N). This distribution pattern bears resemblance to the early stages of Braak NFT staging.^24^ This circuit-aligned accumulation of tau pathology distinguishes rTKhomo mice from rTg4510 mice, despite both utilizing the same promoter. However, it is noteworthy that a similar pathological tau distribution in the entorhinal cortex-hippocampus projection was observed in the rTg TauEC model, where human tau expression is regulated by the Neuropsin promoter.^53^ Hence, rTKhomo mice exhibited the development of region-specific tau pathology despite a broad distribution of tau protein expression across various areas in the forebrain. Furthermore, it is plausible that neurons within the entorhinal cortex-hippocampal projection exhibit heightened susceptibility compared with other regions in the forebrain, indicating regional disparities in vulnerability. Collectively, we have accomplished the development of a model that replicates the location of Alzheimer’s disease-like tau pathology.

### *In vitro* and *in vivo* 3D tau imaging

Whole-brain imaging using tissue-clearing methods and light-sheet microscopy is now becoming widely useable for the profiling of connectivity in the brain.^50,54^ Whole-brain 3D staining with AT8 antibody and light-sheet microscopy is enabled to capture whole-brain pathological tau localization with cellular resolution. In fact, the exact site of tau deposition in the whole brain (Fig. 2) would not have been detectable by conventional immunohistochemistry without the use of 3D imaging. *In vivo*, tau pathology was captured in rTKhomo mice by the PET tracer [^18^F]PM-PBB3. Previous studies reported that *in vivo* visualization of tau pathology was successful in the spinal cords of PS19 and cerebral cortices of rTg4510 mice by tau PET imaging.^34,35,55,56^ Following these reports, the rTKhomo mouse is the third successful model following those two mouse lines. Although the spatial location of PET tracers can be constructed to show a 3D map of pathological tau distribution, the spatial resolution of animal microPET scanner (in the equipment used in this study, <2 mm full-width at half-maximum^57^) is far below light-sheet microscopy. In addition, there is the disadvantage of creating unanalyzable regions due to spillover radioactivity from outside the brain. In fact, some PET tracers accumulate in extracerebral tissues such as the Harderian gland, signal spillover from these sites creates areas where VOI analysis is difficult, and this problem has also been reported in small animal PET using other PET tracers.^58,59^ Due to these factors, *in vitro* and *in vivo* image comparisons did not work well in this study. Importantly, *in vivo* brain imaging enables the longitudinal examination of neurodegenerative processes and pathological tau formation in the same animal.^28,60^ Furthermore, PET imaging analysis is one of the translatable methods between preclinical and clinical research. To make better use of this advantage, whole-brain distributions of PET signals will be precisely evaluated by 3D immunofluorescence imaging in the future.

### Hippocampal tau pathology and glial activation in rTKhomo mice

Spatial distribution of tau pathology in the hippocampus of rTKhomo mice (shown in Figs. 1G, I, 2A and 4A) exhibits notable distinctions from the distributions observed in rTg4510 (Fig. 1H, J) or PS19 mice.^61^ In rTKhomo mice, intraneuronal tau accumulation is primarily localized in CA1, but there are very few in CA2, CA3 and DG (Figs. 1G, I, 5A and 6J, K). In contrast, rTg4510 mice exhibit a more widespread distribution of tau pathology across the entire hippocampus (Fig. 1H, J). Notably, tau pathology in the hippocampus of PS19 mice is less pronounced in the CA1 region^61^ and is comparatively milder than that of rTg4510 mice.^48^ This variance in tau pathology severity may be attributed to the lower level of human tau expression regulated by the prion promoter (∼5-fold overexpression compared with the endogenous mouse tau^8^). It is noteworthy that PS19 mice are widely used for mechanism-based and therapeutic studies.^13,37,55,62-64^ This model recapitulates neurodegenerative phenotypes including formation of NFTs, synaptic dysfunction, cognitive impairment, neuronal loss and gliosis. In contrast to rTKhomo mice, the median life span of the congenic line with C57BL/6J background ranges from 11 to 15 months, primarily due to the onset of limb paralysis.^55^ However, the underlying factors contributing to both the pathological distribution and severity in PS19 mice remain unresolved. On the other hand, since tTA tg mice with a FVB/129 mixed background developed apparent neuronal loss and glial activation in the DG (Fig. 1C–F), pathological tau formation in this area of rTg4510 mice with a FVB/129 background may be caused by tTA toxicity. These tTA tg phenotypes can be eliminated by switching the strain background from FVB/129 to C57BL/6J. Therefore, the reduction of tau pathology in the DG might be feasible if rTg4510 mice could be bred on a C57BL/6J background. However, a previous investigation comparing rTg4510 mice across different backgrounds, specifically FVB/N × 129S6 and FVB/N × C57BL/6NTac, revealed that the rTg4510 mice with a FVB/B6 background exhibited elevated human tau expression at a young age and increased tau phosphorylation at an older age compared with those with a FVB/129 background.^65^ Additionally, hippocampal atrophy was significantly evident in 6.5- and 10.5-month-old rTg4510 mice with a FVB/B6 background, consistent with findings in rTg4510 mice of FVB/129 background.^65^ While a congenic C57BL/6J background ameliorated DG atrophy, the same effect was not observed with B6/C3H or B6/CBA background.^16^ This suggests that a partial C57BL/6J background might not suffice to prevent hippocampal atrophy and tau pathology. Nonetheless, to gain better understanding of the mechanisms of pathological tau formation and neurodegeneration, it is essential to eliminate non-tau factors that could contribute to neuronal impairment. The rTKhomo mouse model was designed to circumvent the uncertain risks associated with non-tau factors such as deletion of *Fgf14* and FVB/129 strain background. Consequently, this model holds promise for providing valuable insights into the interplay between tau protein pathogenesis and neurodegeneration.

In association with pathological tau accumulation, signals of glial markers, IBA1 and GFAP were increased from 12 months of age (Fig. 5). In addition, morphological activation was occurred there (Fig. 6A–D) and we captured a region-specific decrease in the homeostatic microglial marker P2RY12 with the development of tau pathology (Fig. 6J–T). Although the causal relationship between pathological tau accumulation and microglial activation is as yet undefined, slimming of the hippocampal pyramidal layer began at 15 months of age, and this reduction became significant in 18-month-old rTKhomo mice compared with control mice (Fig. 4F and G and Supplementary Fig. 9), suggesting that diminution of homeostatic microglia coincided tau-induced neuronal loss. These data support our previous study of rTg4510 mice showing P2RY12 down-regulation at the early stage of tauopathy.^27^ Furthermore, the reduction of homeostatic microglia was initially observed in the SLM of the hippocampus (Fig. 6K and O). Amoeboid-type microglia was also crowded in this particular region (data not shown). This layer serves as a critical link between the ENT and the CA1 hippocampus.^66^ Therefore, it is plausible that the reduction of homeostatic microglia in the SLM may be triggered by pathological process originating in the ENT. In any case, the regional distribution of tau pathology and the occurrence of neuronal death/microglial response appear to align predominantly in the hippocampal CA1 region of rTKhomo mice. This observation suggests that neurodegeneration and neuroinflammation are intricately linked to the spatiotemporal progression of tau pathology. Nevertheless, further studies using spatially resolved transcriptome analysis and other methods will be needed to demonstrate the morphological and functional spatiotemporal changes in microglial activation.

### Future perspectives of tauopathy mouse models

The unique progression of tau pathology in rTKhomo mice led us to investigate a linkage between tau pathogenesis and cognitive impairments. Since dysfunction of the hippocampus and the entorhinal cortex appeared in mild cognitive impairment and early Alzheimer's disease, pathogenesis of tauopathy in this mouse model may have initiated in these brain regions due to selective vulnerability. While this study primarily focuses on the progression of tau pathology and associated microglial activation, it is essential to elucidate the connection between region-specific tau accumulation and cognitive deficits. Since the accumulation of pathological tau aligned with neural circuits is a unique feature of rTKhomo mice, future investigations should incorporate behavioural studies using this mouse model. Another limitation of this study is its reliance on whole-brain analysis, providing only a single snapshot of tau pathology in 18-month-old rTKhomo mice. This approach restricts our ability to capture the progressive nature of tau accumulation and its variances across different life stages. To address this limitation, future research should encompass broader age range. Such expansion is crucial for enhancing our comprehension of the evolution of tau pathology and its relevance to modeling Alzheimer's Disease.

In conclusion, newly established combination of tg and knock-in mice named rTKhomo mice succeeded in the development of tau pathology. Whole brain 3D staining and light-sheet microscopical imaging revealed the tau pathology along entorhinal-hippocampal connections in rTKhomo mice. Although the natural course of pathogenesis was slow, pathological tau accumulation, neuronal loss and neuroinflammation proceeded with age. Especially in the hippocampal CA1 region, pathological tau deposition and microglial morphological and protein expression changes progressed in concert. The present model will serve as a practical tool for the intervention of tau-induced neurodegeneration and microglia activation.

## Supplementary Material

fcae326_Supplementary_Data

## Data Availability

The authors confirmed that the data supporting the findings of this study are available upon request. The antibodies generated in our laboratory are available to collaborators. The source western blot data for Fig. 1A, Supplementary Figs. 1C–F, 2A, F and 3A, F can be found in Supplementary Figs. 10–12. The codes used in whole brain analysis are available on GitHub at https://github.com/OrganismalSystemsBiology/Tau-analysis.git.
