# Supplementary material for: A novel tauopathy model mimicking molecular and spatial aspects of human tau pathology
Source: Brain Commun. 2024 Sep 19;6(5):fcae326. doi: 10.1093/braincomms/fcae326 (PMC11483584; doi:10.1093/braincomms/fcae326)
Supplement: fcae326_Supplementary_Data [file fcae326_Supplementary_Data.pdf]

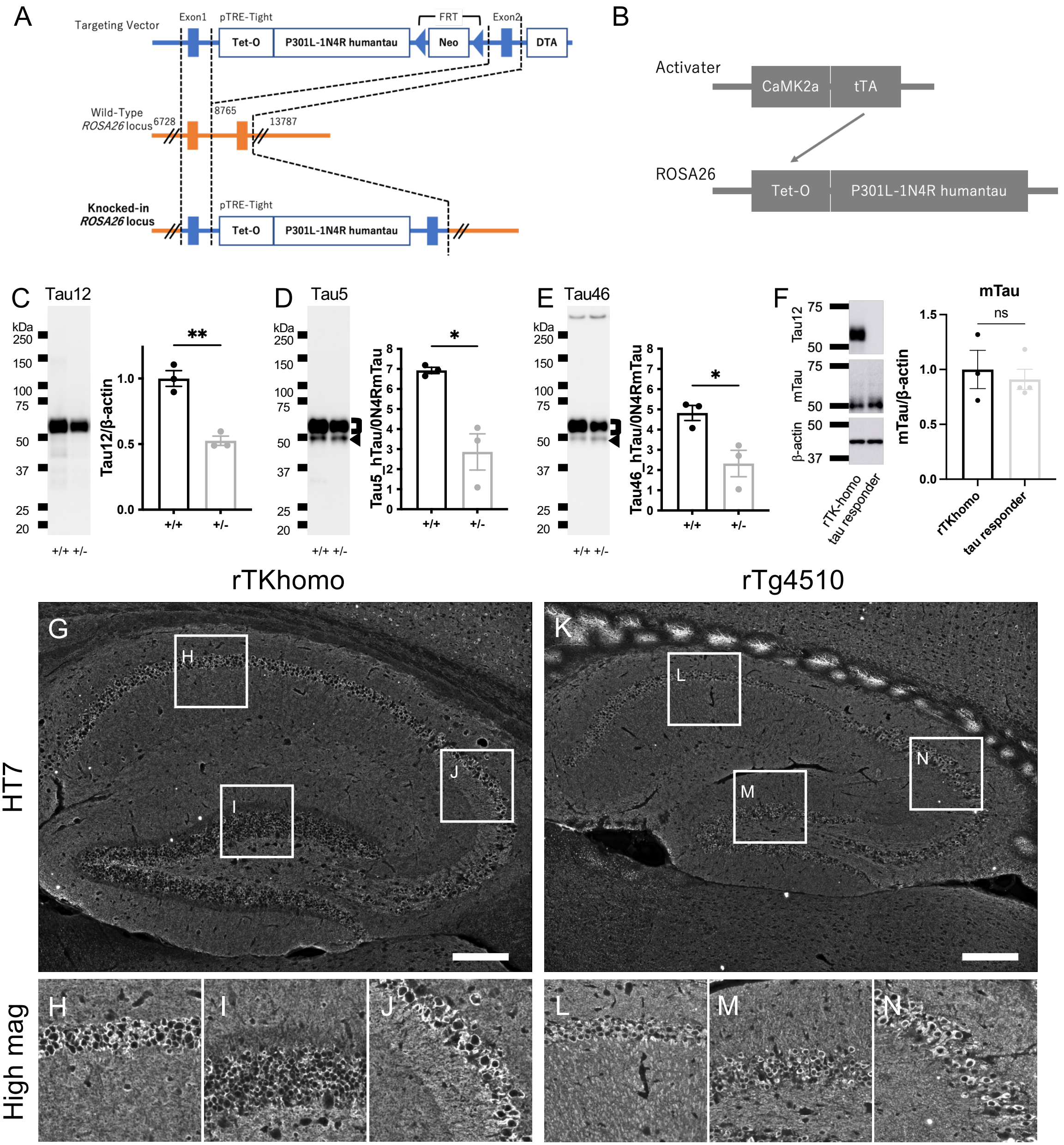

A
Targeting Vector
Exon1
pTRE-Tight
Tet-O
P301L-1N4R humantau
FRT
Neo
Exon2
DTA
Wild-Type ROSA26 locus
6728
8765
13787
Knocked-in ROSA26 locus
B
Activater
CaMK2a
tTA
ROSA26
Tet-O
P301L-1N4R humantau
C
Tau12
Tau12/β-actin
+/+ +/-
D
Tau5
Tau5_hTau/0N4RmTau
E
Tau46
Tau46_hTau/0N4RmTau
F
mTau
ns
mTau/β-actin
rTK-homo
tau responder
rTKhomo
rTg4510
HT7
High mag

**Supplementary Figure 1. Strategy for overexpression of human tau and phosphorylation-independent tau expression analysis.** (A and B) Diagram for knock-in of TRE promoter and P301L mutated human tau into the Rosa26 locus. Green triangles indicate flippase recombinase target sequence. After cross-breeding with FLPe transgenic mouse, the neo caseate was removed in knock-in allele. rTKhetero (tau+/-, tTA+/-) mice were generated by breeding between Tau-KI responder and CaMKII-tTA transgenic mice under C57BL/6 strain background. (C) Quantitative western blot analysis of human tau protein in rTKhomo (tau+/+, tTA+/-) and rTKhetero mice. Representative blotting image (left) and averaged Tau12 levels normalized by β-actin protein levels (right). Values are mean ± SEM. Unpaired t test was performed (**$p<0.01$). (D and E) Quantitative western blot analysis of human tau protein in rTKhomo and rTKhetero mice. Representative blotting image detected by Tau5 (D) and Tau46 (E) antibody (left) and ratio between human tau (1N4R isoform) and mouse tau (0N4R isoform) (right). Values are mean ± SEM. Unpaired t test was performed (*$p<0.05$). Both genotype n=3, 3 males in rTKhomo, 2 males and 1 female in rTKhetero (C-E). (F) Quantitative western blot analysis of mouse tau protein in rTKhomo and tau responder (tau+/+, tTA-/- or tau+/-, tTA-/-) mice. Representative blotting images detected by Tau12 (top), mTau (middle), and β-actin (bottom) and comparison between rTKhomo and tau responder mice (right). rTKhomo (n=3) and tau responder mice (n=4) were examined. Values are mean ± SEM. Unpaired t test was performed. See Supplemental figure 10 for uncropped blots. (G - N) Upper panels show HT7 immunofluorescence staining of hippocampus region from 12-month-old rTKhomo mouse and 3-month-old rTg4510. Inboxes show tau antibody-positive neurons in pyramidal cell layers of CA1 (H and L), CA3 (J and N) and granular cell layer of DG (I and M). Scale bars = 200μm.

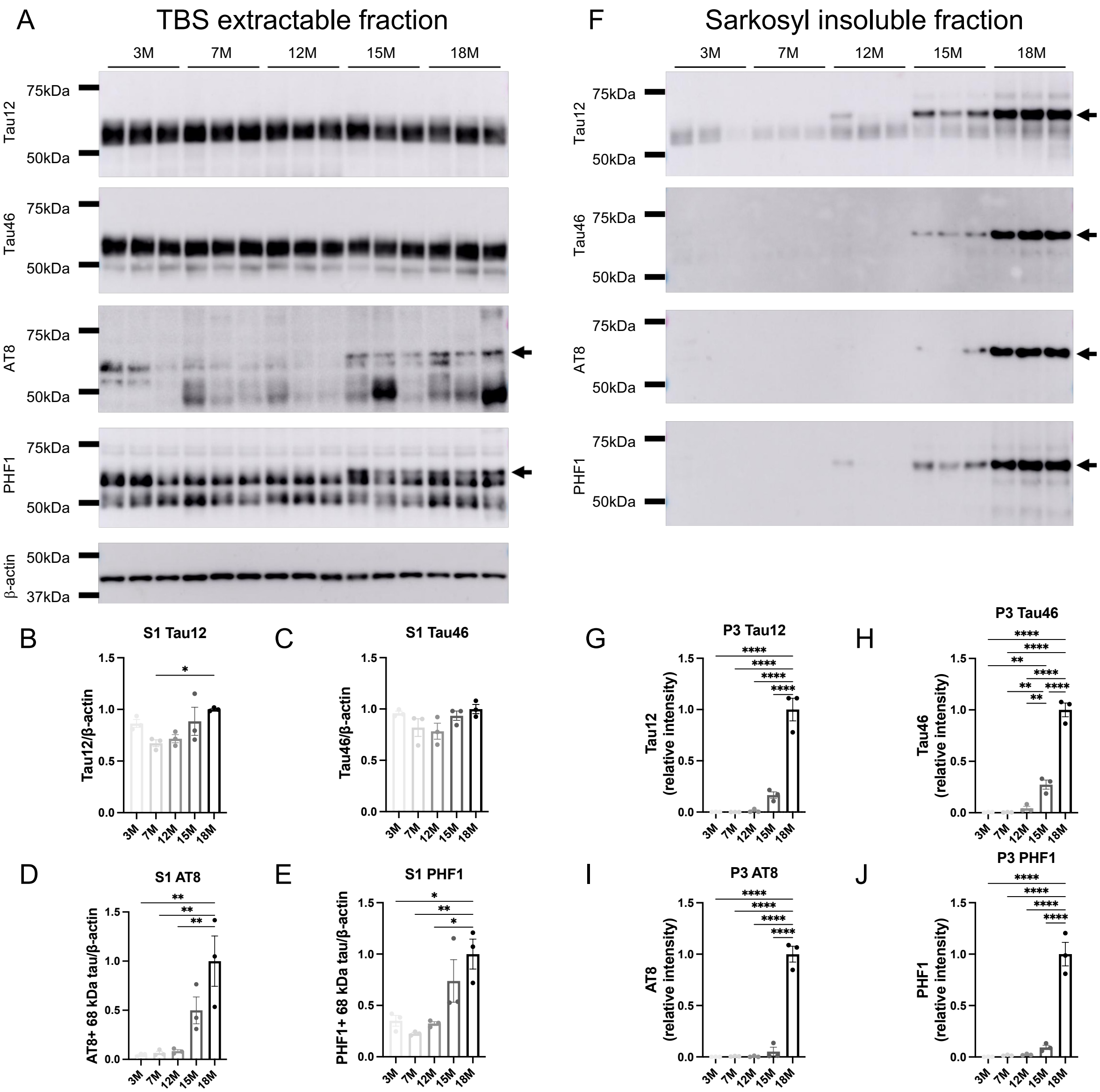

A
TBS extractable fraction
3M 7M 12M 15M 18M
Tau12
Tau46
AT8
PHF1
β-actin
75kDa
50kDa
37kDa
F
Sarkosyl insoluble fraction
B
S1 Tau12
Tau12/β-actin
C
S1 Tau46
Tau46/β-actin
D
S1 AT8
AT8+ 68 kDa tau/β-actin
E
S1 PHF1
PHF1+ 68 kDa tau/β-actin
G
P3 Tau12
Tau12 (relative intensity)
H
P3 Tau46
Tau46 (relative intensity)
I
P3 AT8
AT8 (relative intensity)
J
P3 PHF1
PHF1 (relative intensity)

**Supplementary Figure 2. Tau expression and phosphorylation in TBS-extractable and sarkosyl-insoluble fractions from rTKhomo mouse brains.** (A) Western blots of TBS-extractable fractions from 3- (male, n=3), 7- (male, n=1; female, n=2), 12- (male, n=1; female, n=2), 15- (male, n=1; female, n=2), and 18-month-old (male, n=1; female, n=2) rTKhomo (tau+/+, tTA+/-) mice. Blots were probed with Tau12, Tau46, AT8, PHF1, and b-actin antibodies. Arrows indicate 68 kDa tau. See Supplemental figure 11 for uncropped blots. (B-E) Temporal changes of tau protein levels from 3 to 18 months of age. Relative tau levels detected by Tau12 (B) and Tau46 (C) antibodies were normalized by the levels of β-actin. Relative intensities of 68 kDa bands detected by AT8 (D) and PHF1 (E) were also normalized by the levels of b-actin. Values are mean ± SEM with respect to levels measured from samples of 18-month-old mice; those levels were scaled to a value of one. (F) Western blots of sarkosyl-insoluble fractions from 3-, 7-, 12-, 15-, and 18-month-old rTKhomo mice. Blots were probed with Tau12, Tau46, AT8, and PHF1 antibodies. Arrows indicated 68 kDa tau. See Supplemental figure 11 for uncropped blots. (G-J) Temporal changes of sarkosyl-insoluble tau levels detected by Tau12 (G), Tau46 (H), AT8 (I), and PHF1(J) antibodies from 3 to 18 months of age. Values are mean ± SEM with respect to levels measured from samples of 18-month-old rTKhomo mice; these levels were scaled to a value of one. Tukey's multiple comparisons test was performed (*p<0.05, **p<0.01, ****p<0.0001).

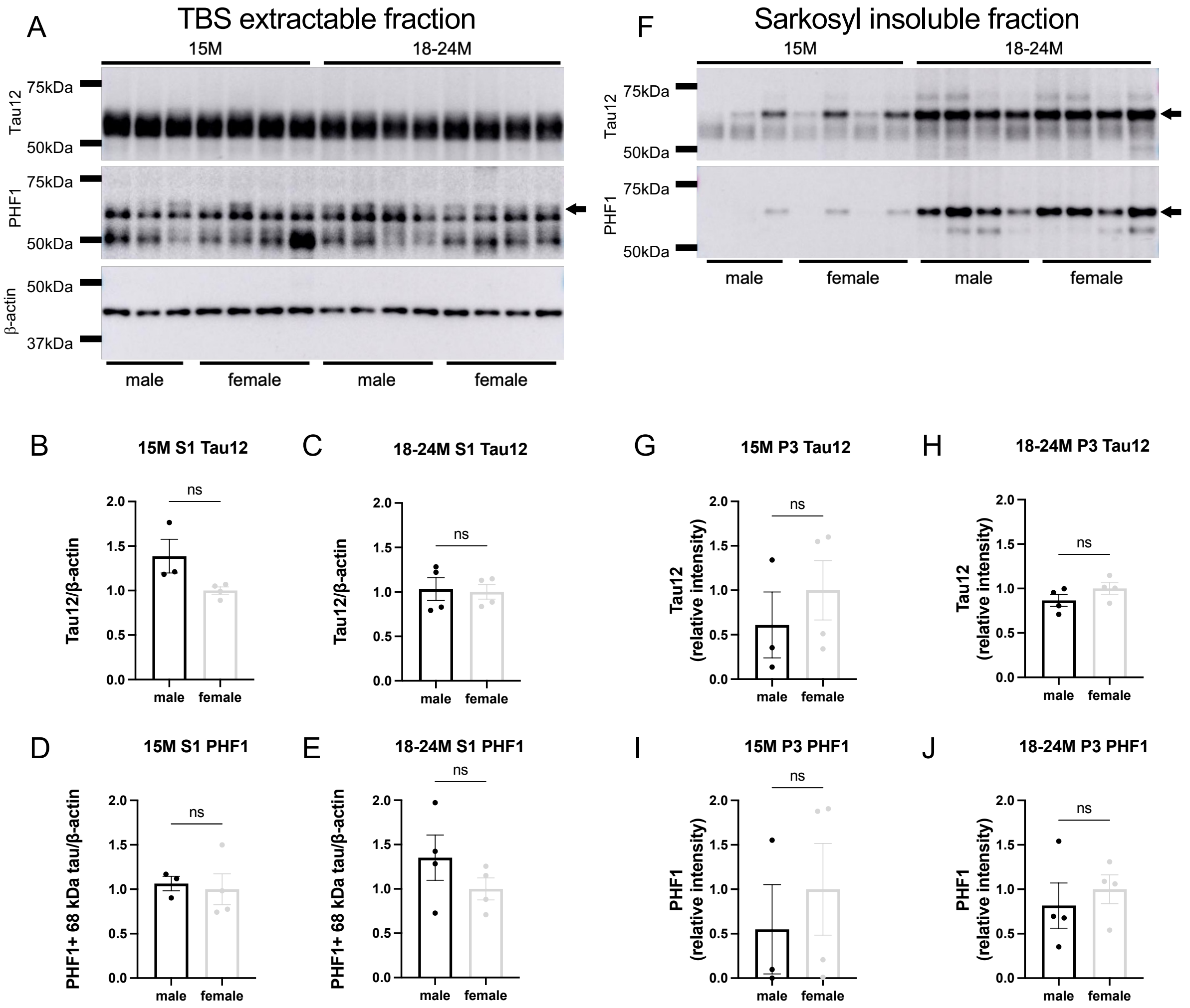


**Supplementary Figure 3. Examination of sex difference in human tau and phosphorylated tau proteins.** (A) Western blots of TBS-extractable fractions from 15- (male, n=3; female, n=4), and 18-24- (male, n=4; female, n=4) rTKhomo (tau+/+, tTA+/-) mice. Blots were probed with Tau12, PHF1, and b-actin antibodies. Arrow indicates 68 kDa tau. See Supplemental figure 12 for uncropped blots. (B-E) Comparison of relative tau levels detected by Tau12 (B, C) and PHF1 (D, E) antibodies were normalized by the levels of β-actin between male and female in 15-month-old (B, D) and 18-24-month-old (C, E) mice. Values are mean ± SEM with respect to levels measured from samples of female mice; those levels were scaled to a value of one. (F) Western blots of sarkosyl-insoluble fractions from 15-, and 18-24-month-old rTKhomo mice. Blots were probed with Tau12, and PHF1 antibodies. Arrow indicates 68 kDa tau. See Supplemental figure 12 for uncropped blots. (G-J) Comparison of relative tau levels detected by Tau12 (G, H) and PHF1 (I, J) antibodies between male and female in 15-month-old (G, I) and 18-24-month-old (H, J) mice. Values are mean ± SEM with respect to levels measured from samples of female mice; those levels were scaled to a value of one. Unpaired t test was performed.

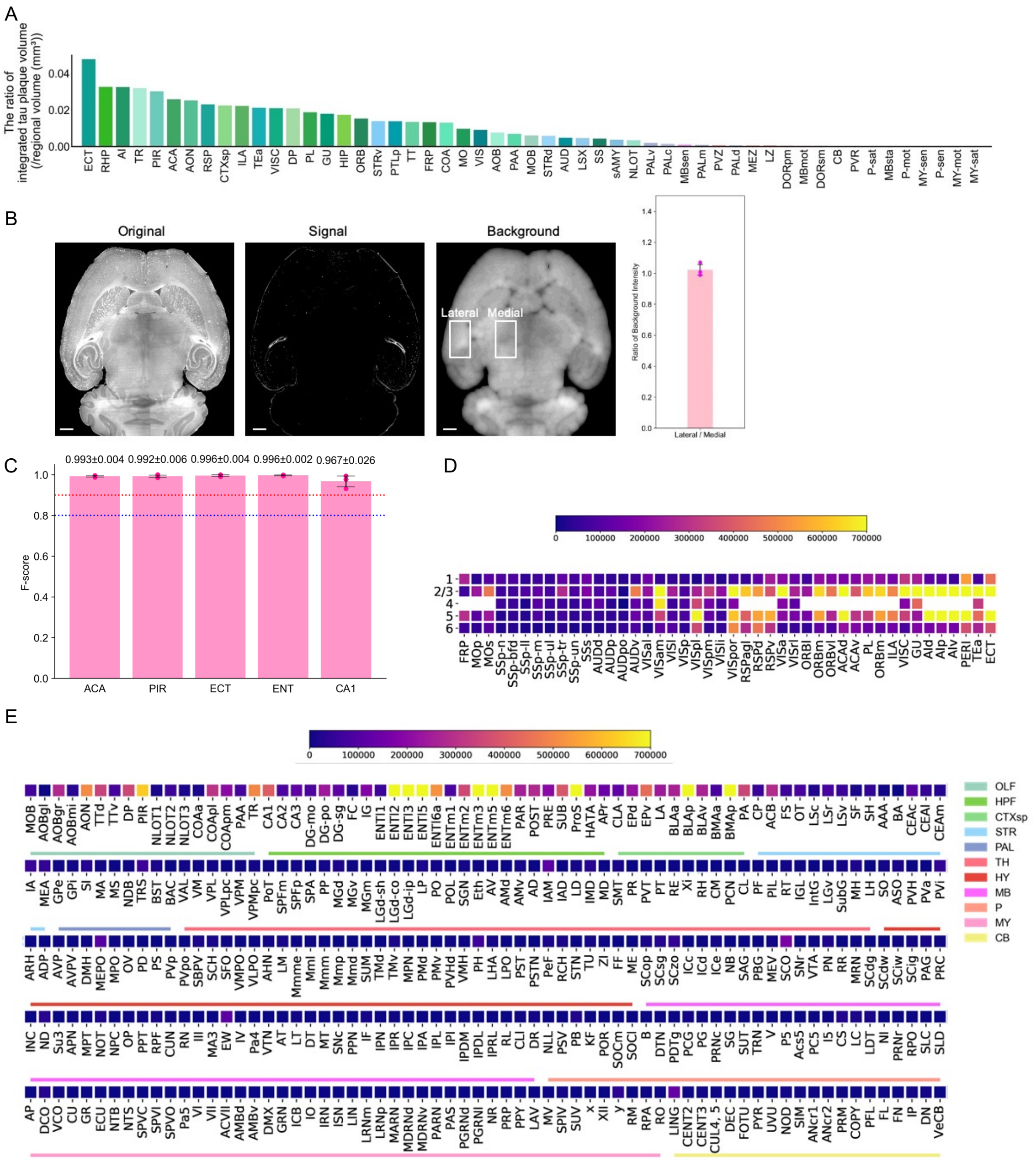

A
The ratio of integrated tau plaque volume (/regional volume (mm³))
ECT RHP AI TR PIR ACA AON RSP CTXsp ILA TEa VISC DP PL GU HIP ORB STRv PTLp TT FRP COA MO VIS AOB PAA MOB STRd AUD LSX SS sAMY NLOT PALv PALc MBsen PALm PVZ PALd MEZ LZ DORpm MBmot DORsm CB PVR P-sat MBsta P-mot MY-sen P-sen MY-mot MY-sat
B
Original
Signal
Background
Lateral
Medial
Ratio of Background Intensity
Lateral / Medial
C
0.993±0.004
0.992±0.006
0.996±0.004
0.996±0.002
0.967±0.026
F-score
ACA
PIR
ECT
ENT
CA1
D
E
OLF
HPF
CTXsp
STR
PAL
TH
HY
MB
P
MY
CB

**Supplementary Figure 4. Accuracy of tau detection analysis and regional heatmap of tau density, related to Figure 2.** (A) Analysis of tau plaque volumes in the whole rTKhomo (tau+/+, tTA+/-) mice brains. The ratio of integrated tau plaque volume per region volume across 53 medium-sized subregions (ref), calculated by summing the volumes of tau deposition spots within each anatomical region and normalizing this sum by the total volume of the region. The ratio is an average derived from data obtained from three 18-month-old rTKhomo mice. (B) Evaluation of light-sheet absorption in whole mouse brains. Representative original, background, and signal images, along with a bar graph that illustrates the ratios of background intensities between manually selected lateral and medial areas for each of the three 18-month-old rTKhomo mice. These ratios were measured within a defined field of 200 $\times$ 350 pixels. A scale bar of 1 mm is included for scale reference. Background images were generated using the 'Subtract Background' function in Fiji software, setting the 'Rolling ball radius' to 70 pixels. Signal images were then produced by subtracting the background images from the original images. (C) Validation of tau detection accuracy across five anatomical regions. F-scores were computed for each of the three 18-month-old rTKhomo mice, using manual ground truths established independently by two experts. The F-scores from the two experts were then averaged to obtain a single score for each sample. The blue line represents an F-score of 0.8, while the red line represents an F-score of 0.9. The values above each bar represent the average of F-score with a 1SD error bar. (D) Average regional tau intensity per volume (/mm3) in the isocortex, calculated from three 18-month-old rTKhomo mice using CUBIC-Cloud software (https://cubic-cloud.com). Density was calculated by dividing the total intensity by the volume of the region. (E) Average regional tau intensity per volume (/mm3), excluding the isocortex, calculated from three 18-month-old rTKhomo mice using CUBIC-Cloud software (https://cubic-cloud.com). The regions are as follows: OLF: olfactory areas, HPF: hippocampal formation, CTXsp: cortical subplate, STR: striatum, PAL: Pallidum, TH: thalamus, HY: hypothalamus, MB: midbrain, P: pons, MY: medulla, CB: cerebellum. Density was calculated by dividing total intensity by volume of the region.

ref: Ying N, Luo H, Li B, et al. Exercise Alleviates Behavioral Disorders but Shapes Brain Metabolism of APP/PS1 Mice in a Region- and Exercise-Specific Manner. J Proteome Res. Jun 2 2023;22(6):1649-1659. doi:10.1021/acs.jproteome.2c00691

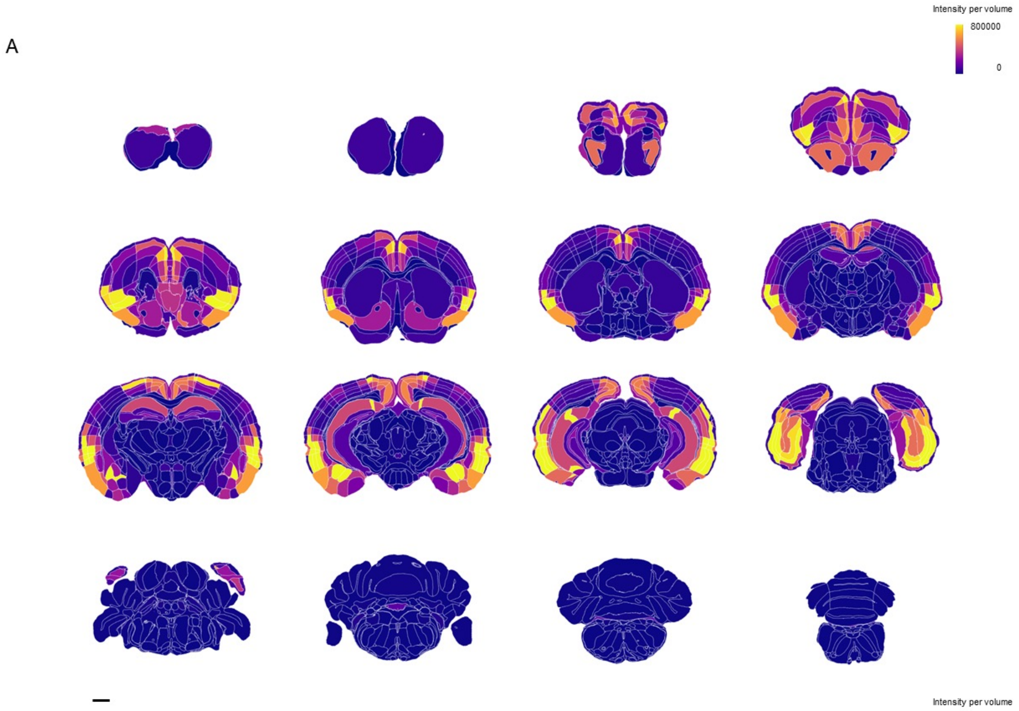


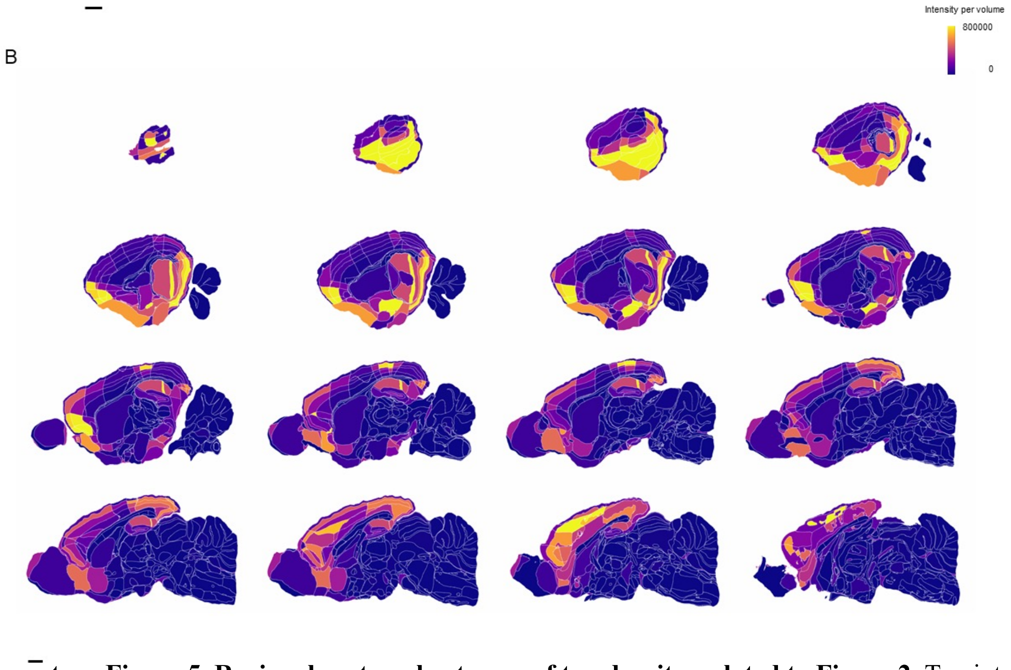


**Supplementary Figure 5. Regional cartoon heatmaps of tau density, related to Figure 2.** Tau intensity per volume (/mm3) was calculated by dividing the total intensity of tau by the volume of the region. (A) Cartoon heatmap of average regional tau intensity per volume in coronal slices, derived from the data of three 18-month-old rTKhomo (tau+/+, tTA+/-) mice using CUBIC-Cloud software (https://cubic-cloud.com). Scale: 2 mm. (B) Cartoon heatmap of average regional tau intensity per volume in sagittal slices, derived from the data of three 18-month-old rTKhomo mice using CUBIC-Cloud software (https://cubic-cloud.com). Scale: 2 mm.

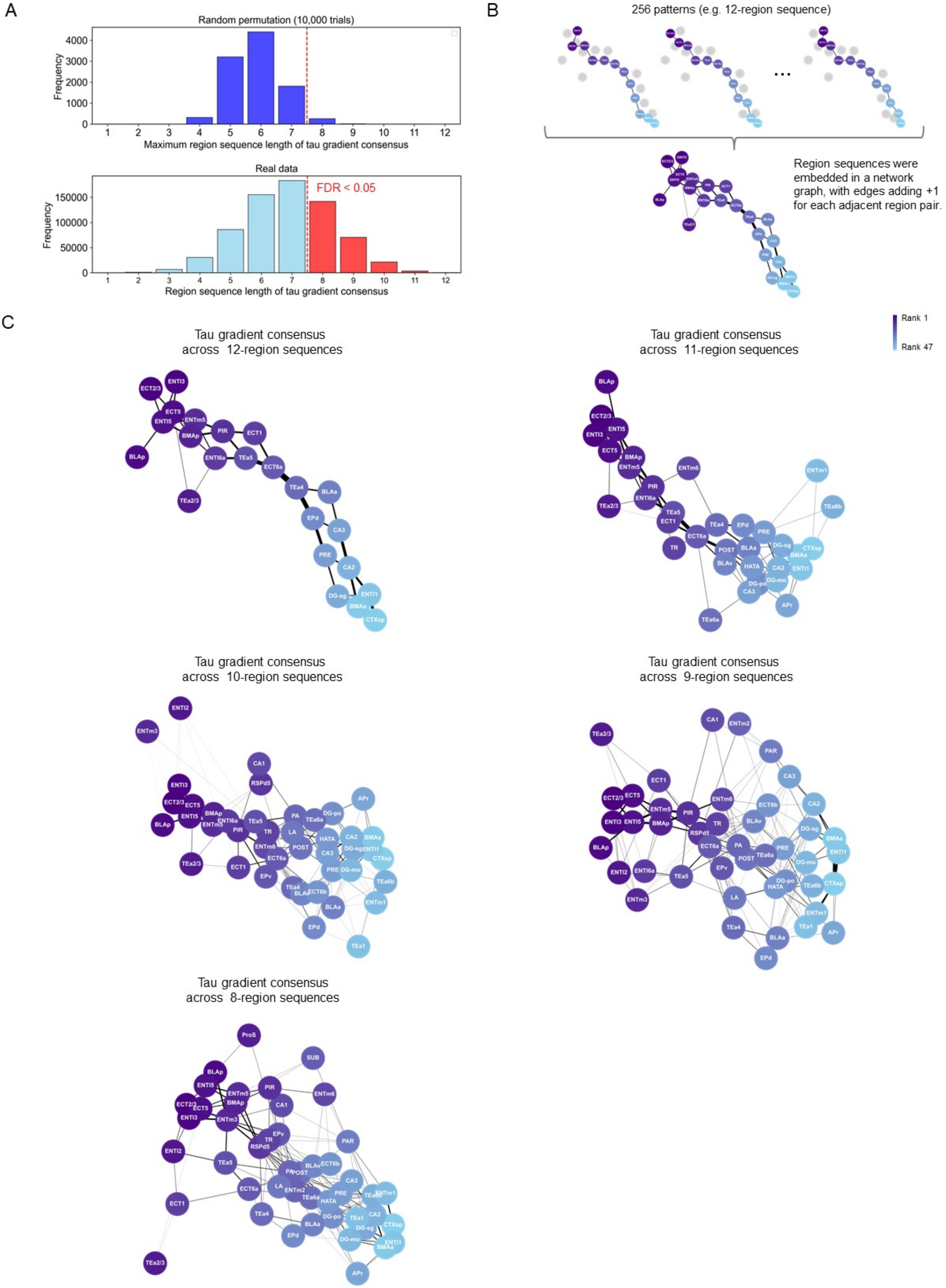
A
Random permutation (10,000 trials)
Frequency
Maximum region sequence length of tau gradient consensus
Real data
FDR < 0.05
Region sequence length of tau gradient consensus
B
256 patterns (e.g. 12-region sequence)
Region sequences were embedded in a network graph, with edges adding +1 for each adjacent region pair.
C
Rank 1
Rank 47
Tau gradient consensus across 12-region sequences
Tau gradient consensus across 11-region sequences
Tau gradient consensus across 10-region sequences
Tau gradient consensus across 9-region sequences
Tau gradient consensus across 8-region sequences

**Supplementary Figure 6. Summary of tau gradient consensus analysis, related to Figure 2.** (A) Determination of the statistical threshold for tau gradient consensus analysis using a False Discovery Rate (FDR) approach. The upper panel shows a histogram of maximum region lengths across 10,000 random permutation trials. A significant threshold of length 8 was established based on the FDR, where less than 5% of trials had a region of that size or larger. The lower panel shows the distribution of region lengths from the real data of three 18-month-old rTKhomo (tau+/+, tTA+/-) mice, with significant lengths highlighted in red. For methodological details, see "Workflow of tau gradient consensus analysis" in the Materials and methods. (B) A scheme of summarizing region sequences to a graph network. One adjacent region pair was described as an edge linking the corresponding region node. For example, 12-region sequences were embedded in a network graph, with edges adding +1 for each adjacent region pair. (C) Network graphs were generated for each length, encapsulating the consensus on the tau gradient for lengths ranging from 8 to 12. Given that the sequences of higher lengths invariably include those of the previous length, we selected exclusive sequences in a descending order from 12, ensuring they were not entirely identical to sequences of higher lengths. Meanwhile, sequences with partial matches were retained. Edge weights were initially computed by incrementing the adjacency values among patterns of identical regional length by one. These weights were subsequently normalized to ensure that the total sum of all weights equaled 250. The color coding of the nodes was determined based on the rank of the mean regional tau density, arranged in descending order.

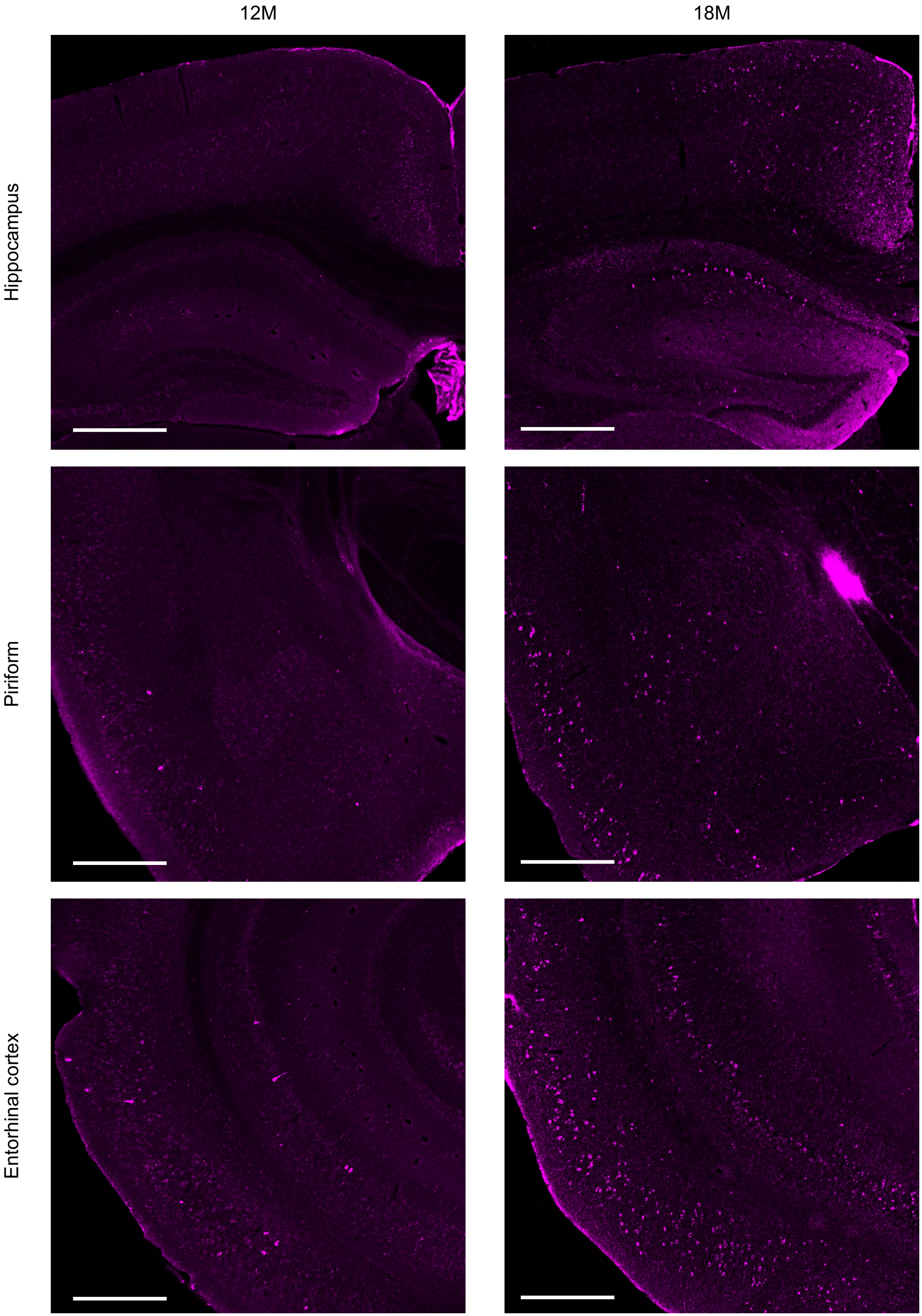


**Supplemental figure 7. 2D-coronal AT8 staining of 12- and 18-month-old rTKhomo mice.** Representative AT8 immunofluorescence-stained images in hippocampus, piriform and entorhinal cortex of 12- and 18-month-old rTKhomo (tau+/+, tTA+/-) mice. Scale bars = 500μm.

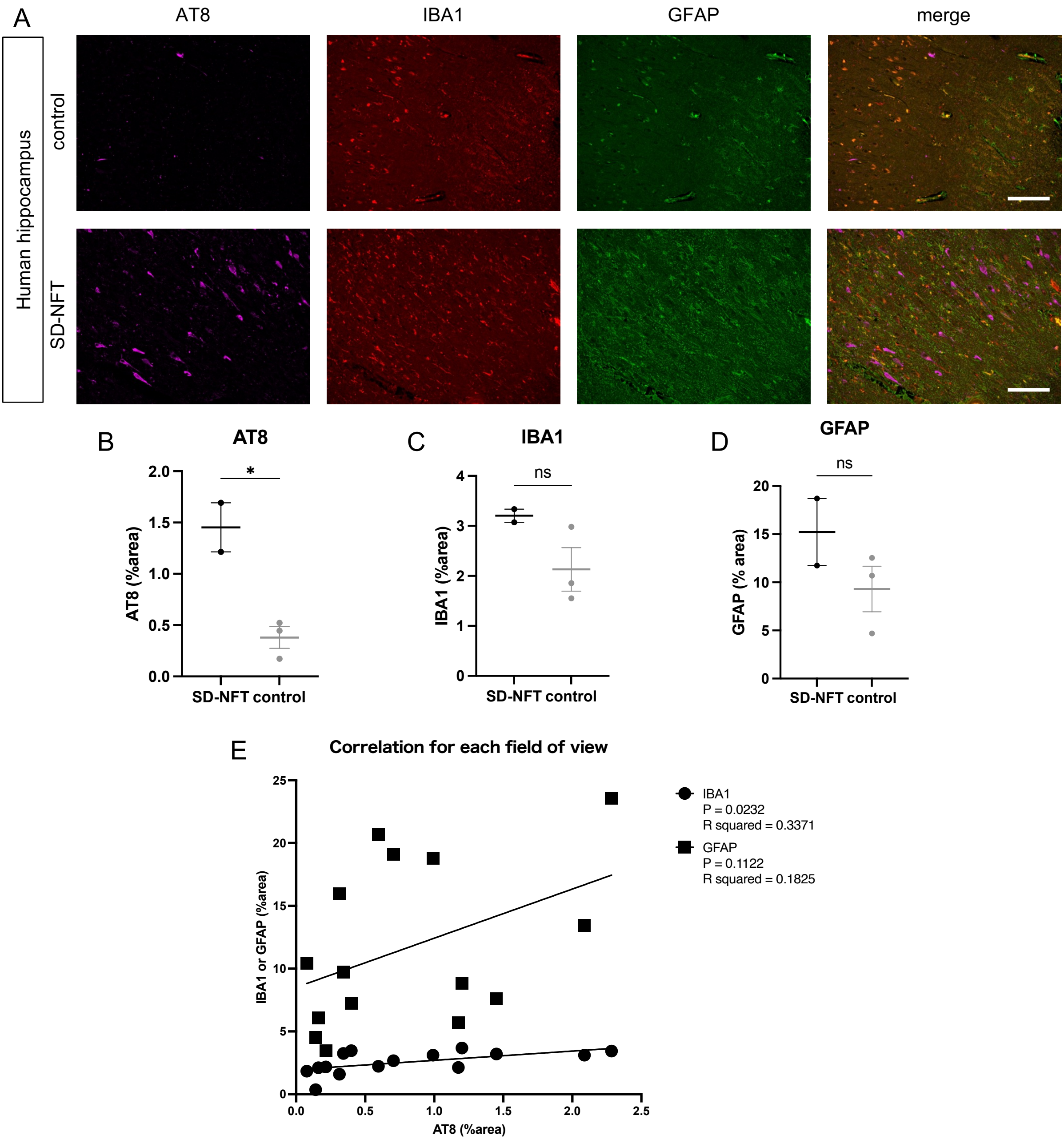


**Supplementary Figure 8. Activation of microglia and astrocytes in human brain subjects.** (A) Representative AT8, IBA1 and GFAP immunofluorescence-stained images in hippocampus in senile dementia of the NFT type (SD-NFT) patient and non-demented healthy control. Scale bars = 100μm. (B-D) Semi-quantification of AT8 (B), IBA1(C), and GFAP signals in hippocampus of SD-NFT patient (female, n=2) and non-demented healthy control (female, n=2; male, n=1). Values are mean ± SEM. Unpaired t test was performed (*p<0.05) (E) Scatterplots of IBA1 and GFAP immunoreactivities in hippocampi for AT8-signals. Pearson correlation coefficient showed a significant correlation in IBA1 signals (p=0.0232; R squared = 0.3371).

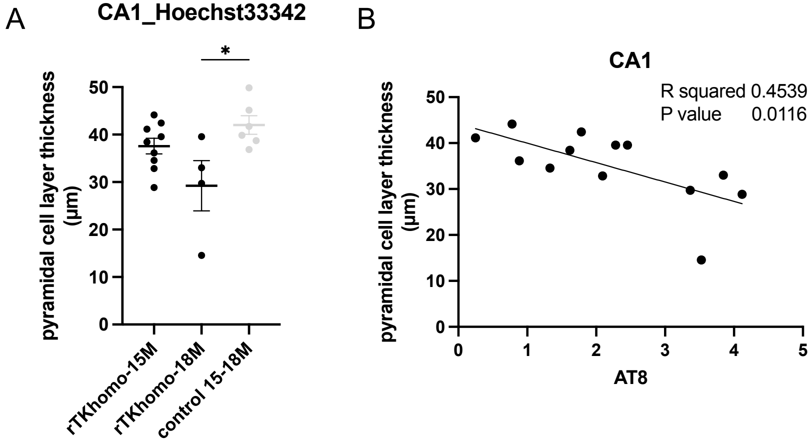


**Supplementary Figure 9. Analysis of pyramidal cell layer thickness in the hippocampal CA1.** Hoechest33342-labeled hippocampal CA1area was measured for fluorescence signals by BZ-X Analyzer. Thickness of the cell layer was quantitated with vertical lines drawn from the outside of each point (CA1; 7 points). Cell layer thickness of each region was calculated from the average of each point. (A) Thicknesses of CA1 pyramidal cell layer in 15-month-old rTKhomo (male, n=6; female, n=3), 18-month-old rTKhomo (female, n=4) and 15-18-month-old control (male, n=4; female, n=2) mice. Thickness values are mean ± SEM. Tukey’s multiple comparison test was performed (*p<0.05). (B) Scatterplots of CA1 pyramidal cell layer thickness for AT8-signals in 15-18-month-old rTKhomo (male, n=6; female, n=7). Pearson correlation coefficient showed a significant correlation (p=0.0116; R squared = 0.4539).

Fig. 1A

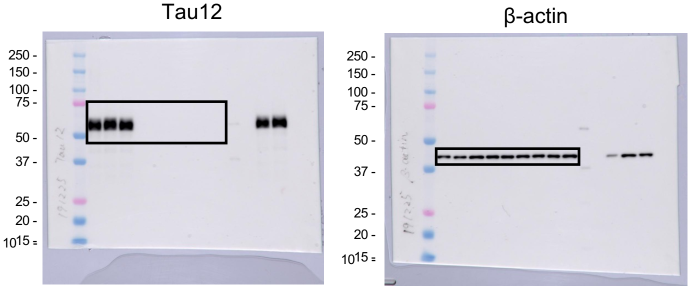


Supplemental Fig. 1C-E

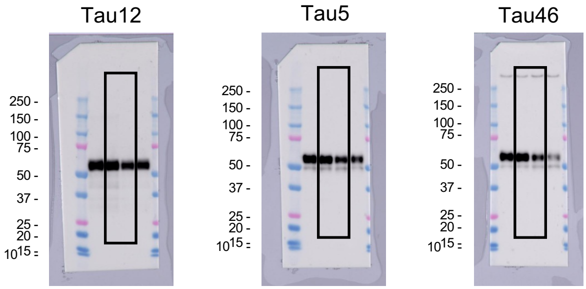


Supplemental Fig. 1F

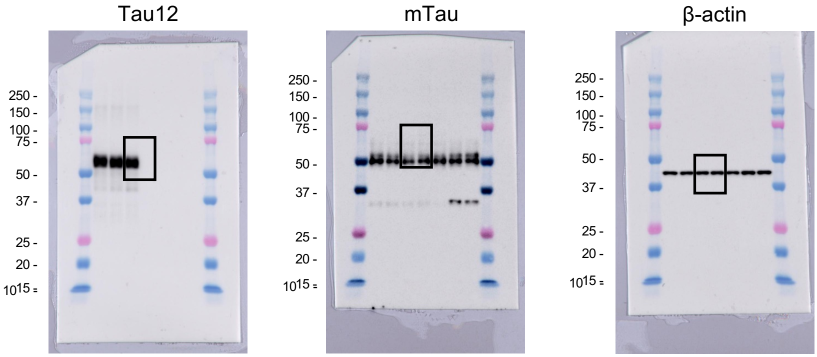


**Supplementary Figure 10. Full size images for western blot shown in Fig. 1A, Supplemental Fig. 1C-F.** The black box indicates the trimming area used in the figures.

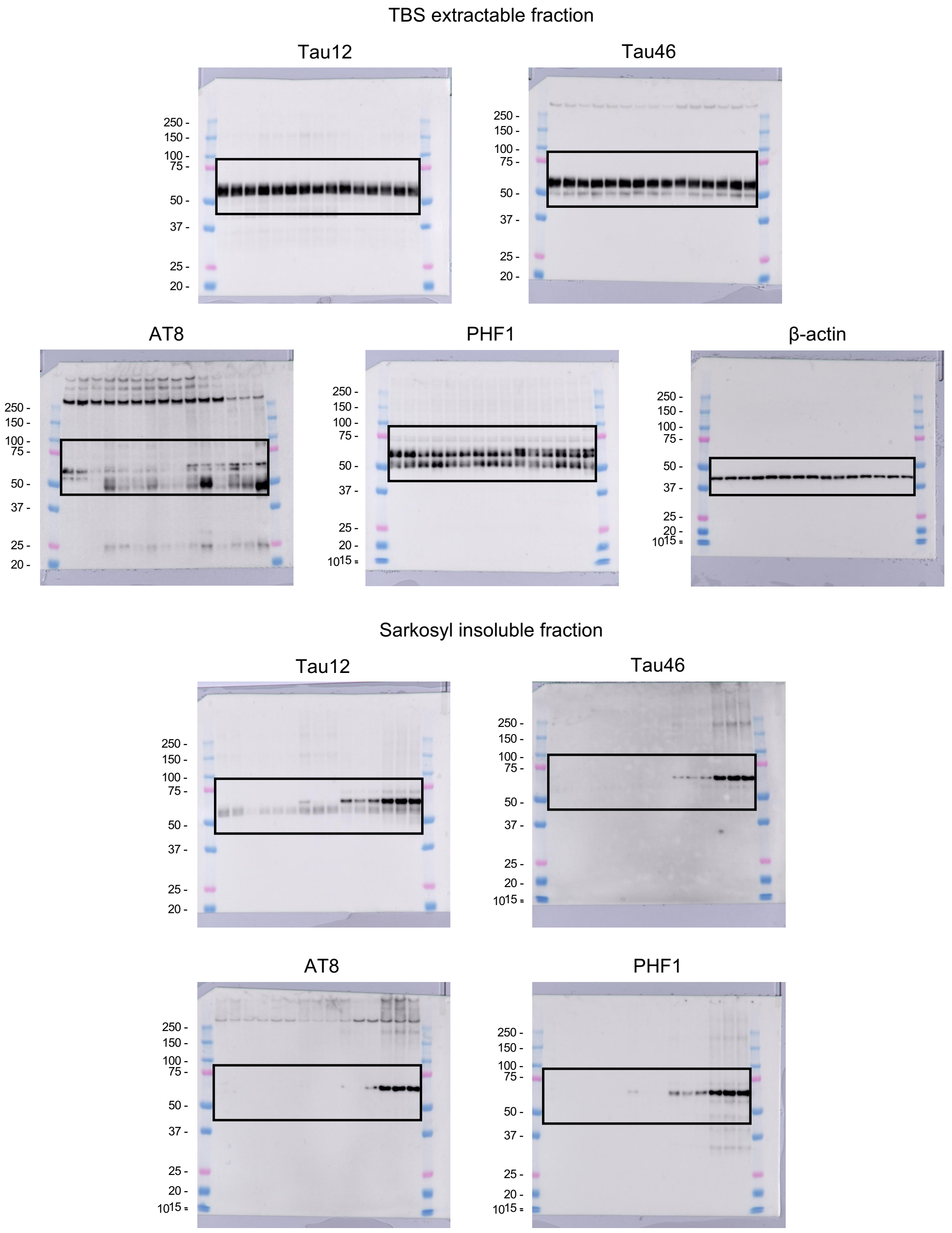


**Supplementary Figure 11. Full size images for western blot shown in Supplemental Fig. 2A, F.** The black box indicates the trimming area used in the figure.

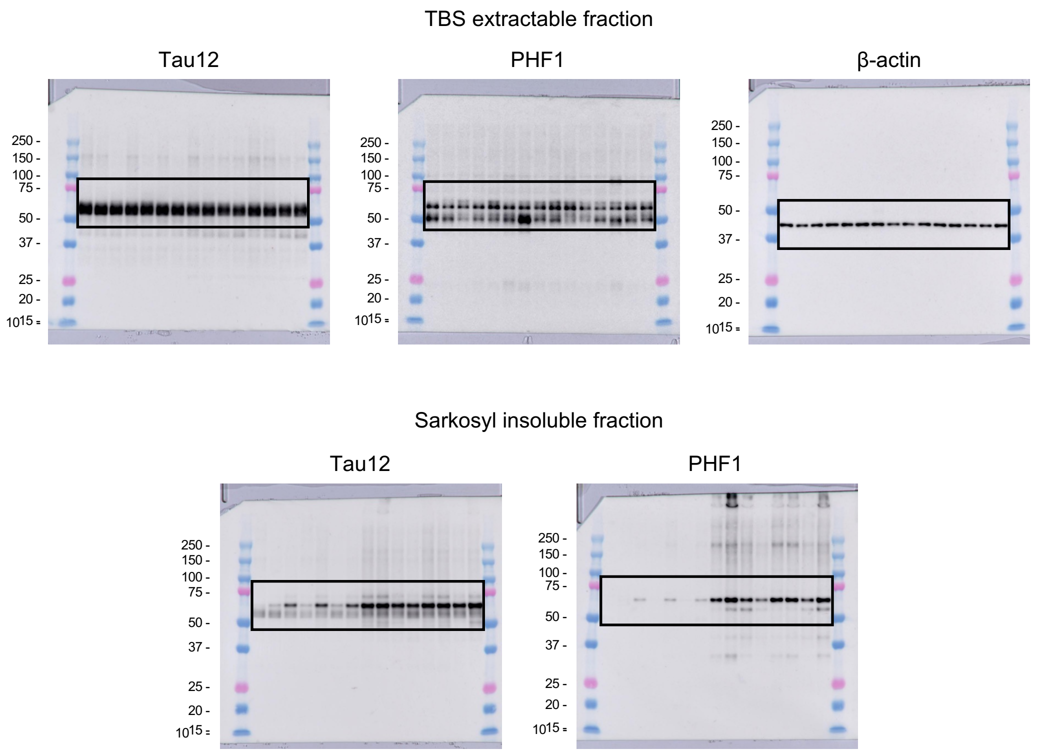


**Supplementary Figure 12. Full size images for western blot shown in Supplemental Fig. 3A, F.** The black box indicates the trimming area used in the figure.

**Supplemental table 1: List of all experimental animals**

| | | | *Genotype* | | | |
|---|---|---|---|---|---|---|
| *ID* | *Age (month)* | *Sex* | *Tau* | *tTA* | *Generation* | *Figures* |
| 2 | 2M | F | +/- | + | 1 | Fig. 1A, B, Sup. 1C-E |
| 26 | 2M | M | +/- | + | 2 | Fig. 1A, B, Sup. 1C-E |
| 27 | 2M | M | +/- | + | 2 | Fig. 1A, B, Sup. 1C-E |
| 7 | 2M | M | +/- | - | 1 | Fig. 1A, B, Sup. 1F |
| 12 | 2M | M | +/- | - | 1 | Fig. 1A, B |
| 13 | 2M | M | +/- | - | 1 | Fig. 1A, B, Sup. 1F |
| 4 | 2M | F | - | - | 1 | Fig. 1A, B |
| 6 | 2M | M | - | - | 1 | Fig. 1A, B |
| 8 | 2M | M | - | - | 1 | Fig. 1A, B |
| 423 | 2M | F | - | - | 7 | Fig. 1C, D |
| 425 | 2M | F | - | - | 7 | Fig. 1C, D |
| 427 | 2M | F | - | - | 7 | Fig. 1C, D |
| 424 | 2M | F | - | + | 7 | Fig. 1C, D |
| 426 | 2M | F | - | + | 7 | Fig. 1C, D |
| 428 | 2M | F | - | + | 7 | Fig. 1C, D |
| 362 | 3M | M | +/+ | + | 5 | Sup. 1C-E, 2, Fig. 3D, Fig. 4 |
| 363 | 3M | M | +/+ | + | 5 | Sup. 1C-E, 2, Fig. 3D, Fig. 4 |
| 374 | 3M | M | +/+ | + | 5 | Sup. 1C-E, 2, Fig. 3D, Fig. 4 |
| 404 | 3M | F | +/+ | + | 6 | Fig. 3D |
| 405 | 3M | F | +/+ | + | 6 | Fig. 3D |
| 407 | 3M | F | +/+ | + | 6 | Fig. 3D |
| 371 | 3M | M | +/+ | - | 5 | Fig. 3D, Fig. 4 |
| 372 | 3M | M | +/+ | - | 5 | Fig. 3D, Fig. 4 |
| 292 | 7M | F | +/+ | + | 4 | Sup. 2, Fig. 3D, Fig. 4 |
| 293 | 7M | F | +/+ | + | 4 | Sup. 2, Fig. 3D, Fig. 4 |
| 296 | 7M | M | +/+ | + | 4 | Sup. 2, Fig. 3D, Fig. 4 |
| 369 | 7M | F | +/+ | + | 5 | Fig. 3D, Fig. 4 |
| 360 | 7M | M | +/+ | - | 5 | Fig. 4 |
| 361 | 7M | M | +/+ | - | 5 | Fig. 4 |
| 364 | 7M | M | +/+ | - | 5 | Fig. 4 |
| 245 | 12M | F | +/+ | + | 4 | Sup. 2, Fig. 3D, Fig. 4 |
| 246 | 12M | F | +/+ | + | 4 | Sup. 2, Fig. 3D, Fig. 4 |
| 247 | 12M | M | +/+ | + | 4 | Sup. 2, Fig. 3D, Fig. 4 |
| 282 | 12M | F | +/+ | + | 3 | Sup. 1G-J |
| 543 | 12M | F | +/+ | + | 7 | Sup. 7 |

| | | | | | | |
|---|---|---|---|---|---|---|
| 242 | 12M | F | +/+ | - | 4 | Fig. 3D, Fig. 4 |
| 243 | 12M | F | +/+ | - | 4 | Fig. 3D, Fig. 4 |
| 244 | 12M | F | +/+ | - | 4 | Fig. 3D, Fig. 4 |
| 224 | 15M | F | +/+ | + | 3 | Sup. 3, Fig. 3D, Fig. 4, Fig. 5J-T, Sup. 9A, B |
| 225 | 15M | F | +/+ | + | 3 | Sup. 2, Sup. 3, Fig. 3D, Fig. 4, Fig. 5J-T, Sup. 9A, B |
| 226 | 15M | M | +/+ | + | 3 | Sup. 3, Fig. 3D, Fig. 4, Fig. 5J-T, Sup. 9A, B |
| 264 | 15M | F | +/+ | + | 4 | Sup. 3, Fig. 3D |
| 267 | 15M | F | +/+ | + | 4 | Sup. 2, Sup. 3, Fig. 3D, Fig. 4, Fig. 5J-T, Sup. 9A, B |
| 280 | 15M | M | +/+ | + | 4 | Sup. 3, Fig. 3D, Fig. 4, Fig. 5J-T, Sup. 9A, B |
| 281 | 15M | M | +/+ | + | 4 | Sup. 2, Sup. 3, Fig. 3D, Fig. 4, Fig. 5J-T, Sup. 9A, B |
| 284 | 15M | M | +/+ | + | 4 | Fig. 5J-T, Sup. 9A, B |
| 285 | 15M | M | +/+ | + | 4 | Fig. 5J-T, Sup. 9A, B |
| 286 | 15M | M | +/+ | + | 4 | Fig. 5J-T, Sup. 9A, B |
| 227 | 15M | M | +/+ | - | 3 | Fig. 3D, F-I, Fig. 4, Sup. 9A |
| 287 | 15M | M | +/+ | - | 4 | Fig. 3F-I, Fig. 4, Sup. 9A |
| 288 | 15M | M | +/+ | - | 4 | Fig. 3F-I, Fig. 4, Sup. 9A |
| 229 | 18M | F | +/+ | + | 3 | Fig. 3D, F-I, Fig. 4, Fig. 5A-I |
| 238 | 18M | M | +/+ | + | 4 | Fig. 1G, I, Sup. 2, Sup. 3, Fig. 3D, E, F-I, Fig. 5A-I |
| 239 | 18M | M | +/+ | + | 4 | Fig. 3E |
| 240 | 18M | F | +/+ | + | 4 | Fig. 3D, E, F-I, Fig. 4, Fig. 5A-I |
| 248 | 18M | M | +/+ | + | 4 | Fig. 3E |
| 250 | 18M | F | +/+ | + | 4 | Sup. 2, Sup. 3, Fig. 3D, E, F-I, Fig. 4, Fig. 5J-T, Fig. 5A-I, Sup. 9A, B |
| 255 | 18M | F | +/+ | + | 4 | Sup. 2, Sup. 3, Fig. 3D, E, F-I, Fig. 5J-T, Sup. 9A, B |
| 298 | 18M | F | +/+ | + | 4 | Fig. 3F-I, Fig. 4, Fig. 5J-T, Fig. 5A-I, Sup. 9A, B |
| 299 | 18M | F | +/+ | + | 4 | Fig. 3F-I, Fig. 4, Fig. 5J-T, Fig. 5A-I, Sup. 9A, B |
| 351 | 18M | M | +/+ | + | 5 | Fig. 2 |
| 356 | 18M | M | +/+ | + | 5 | Fig. 2 |
| 357 | 18M | M | +/+ | + | 5 | Fig. 2 |
| 336 | 18M | F | +/+ | + | 5 | Sup. 7 |
| 358 | 18M | F | +/+ | + | 5 | Fig. 3A-C |
| 359 | 18M | F | +/+ | + | 5 | Fig. 3A-C |
| 368 | 18M | F | +/+ | + | 5 | Fig. 3A-C |
| 523 | 18M | M | +/+ | + | 6 | Sup. 7 |
| 236 | 18M | F | +/+ | - | 4 | Fig. 3D, E, F-I |
| 241 | 18M | M | +/+ | - | 4 | Fig. 3D, E, F-I, Fig. 4, Fig. 5A-I, Sup. 9A |
| 249 | 18M | F | +/+ | - | 4 | Fig. 3D, E |
| 251 | 18M | F | +/+ | - | 4 | Fig. 3D, E |
| 258 | 18M | M | +/+ | - | 4 | Fig. 3D, E |

| | | | | | | |
|---|---|---|---|---|---|---|
| 259 | 18M | M | +/+ | - | 4 | Fig. 3E |
| 297 | 18M | F | +/+ | - | 4 | Fig. 4, Fig. 5A-I, Sup. 9A |
| 300 | 18M | F | +/+ | - | 4 | Fig. 4, Fig. 5A-I, Sup. 9A |
| 366 | 18M | F | +/+ | - | 5 | Fig. 3A-C |
| 367 | 18M | F | +/+ | - | 5 | Fig. 3A-C |
| 379 | 18M | F | +/+ | - | 5 | Fig. 3A-C |
| 521 | 18M | M | +/+ | - | 6 | Sup. 7 |
| 450 | 24M | M | +/+ | + | 6 | Sup. 3 |
| 451 | 24M | M | +/+ | + | 6 | Sup. 3 |
| 454 | 24M | F | +/+ | + | 6 | Sup. 3 |
| 455 | 24M | F | +/+ | + | 6 | Sup. 3 |
| 460 | 24M | M | +/+ | + | 6 | Sup. 3 |
| | | | | | | |
| rTg4510 | | | | | | |
| 761.1 | 2M | F | - | - | | Fig. 1C, D |
| 763.1 | 2M | F | - | - | | Fig. 1C, D |
| 765.1 | 2M | F | - | - | | Fig. 1C, D |
| 761.3 | 2M | F | - | + | | Fig. 1C, D |
| 763.4 | 2M | F | - | + | | Fig. 1C, D |
| 764.1 | 2M | F | - | + | | Fig. 1C, D |
| 670.2 | 3M | F | +/- | + | | Sup. 1K-N |
| 620.13 | 6M | M | +/- | + | | Fig. 1H, J, Fig. 5A-I |
| 621.1 | 6M | M | +/- | + | | Fig. 5A-I |
| 627.1 | 7M | F | +/- | + | | Fig. 5A-I |
| 627.3 | 7M | F | +/- | + | | Fig. 5A-I |
| 830.1 | 7M | F | +/- | + | | Fig. 5A-I |

F: femal, M: male
